# Predicting biochemical oxygen demand in European freshwater bodies

**DOI:** 10.1016/j.scitotenv.2019.02.252

**Published:** 2019-05-20

**Authors:** Olga Vigiak, Bruna Grizzetti, Angel Udias-Moinelo, Michela Zanni, Chiara Dorati, Fayçal Bouraoui, Alberto Pistocchi

**Affiliations:** a European Commission, Joint Research Centre (JRC), Ispra, Italy; b Ludwig-Maximilians-Universitaet Muenchen, Department of Geography, Munich, Germany

**Keywords:** BOD, Green^+^, Water quality, Organic pollution, Water Framework Directive

## Abstract

Biochemical Oxygen Demand (BOD) is an indicator of organic pollution in freshwater bodies correlated to microbiological contamination. High BOD concentrations reduce oxygen availability, degrade aquatic habitats and biodiversity, and impair water use. High BOD loadings to freshwater systems are mainly coming from anthropogenic sources, comprising domestic and livestock waste, industrial emissions, and combined sewer overflows. We developed a conceptual model (GREEN^+^_BOD_) to assess mean annual current organic pollution (BOD fluxes) across Europe. The model was informed with the latest available European datasets of domestic and industrial emissions, population and livestock densities. Model parameters were calibrated using 2008–2012 mean annual BOD concentrations measured in 2157 European monitoring stations, and validated with other 1134 stations. The most sensitive model parameters were abatement of BOD by secondary treatment and the BOD decay exponent of travel time. The mean BOD concentrations measured in monitored stations was 2.10 mg O_2_/L and predicted concentrations were 2.54 mg O_2_/L; the 90th percentile of monitored BOD concentration was 3.51 mg O_2_/L while the predicted one was 4.76 mg O_2_/L. The model could correctly classify reaches for BOD concentrations classes, from high to poor quality, in 69% of cases. High overestimations (incorrect classification by 2 or more classes) were 2% and large underestimations were 5% of cases. Across Europe about 12% of freshwater network was estimated to be failing good quality due to excessive BOD concentrations (>5 mg O_2_/L). Dominant sources of BOD to freshwaters and seas were point sources and emissions from intensive livestock systems. Comparison with previous assessments confirms a decline of BOD pollution since the introduction of EU legislation regulating water pollution.

## Highlights

• A new BOD conceptual model was developed using the most updated datasets.
• Model parameters could be well identified using European BOD monitoring network.
• Organic pollution (BOD > 5 mg/L) still affects about 14% of European surface waters.
• In North Europe, livestock waste contributed 38–49% of BOD loads to the sea.
• Domestic waste was the major source of BOD to the Mediterranean and Black seas.

## Graphical abstract

**Figure:**
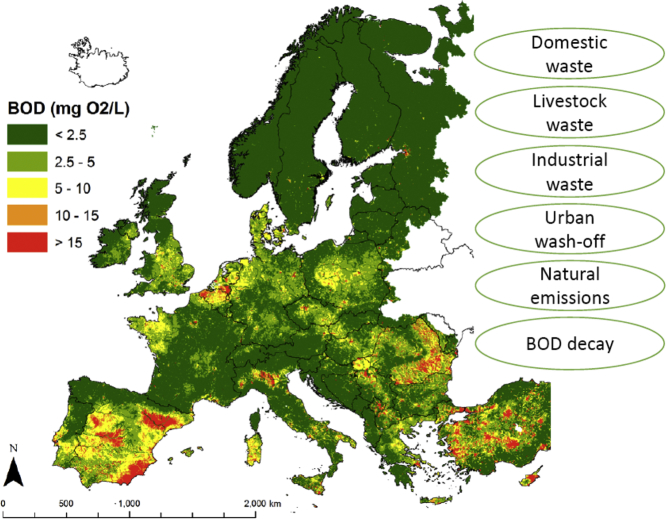

## 1 Introduction

Biochemical Oxygen Demand (BOD) is the amount of oxygen used by organisms to consume oxidisable organic matter in a given time (EEA, 2015). BOD is an indicator of organic pollution in freshwater bodies (Vörösmarty et al., 2010; Wen et al., 2017), correlated to microbiological contamination. High BOD concentrations reduce oxygen availability, degrade aquatic habitats and biodiversity (Ferreira et al., 2017), and impair water use. High BOD loadings to freshwater systems are mainly coming from anthropogenic sources, comprising domestic and livestock waste, industrial emissions, and combined sewer overflows. While transported through the stream network, BOD concentrations are reduced by microbial degradation (river self-purification) and dilution before reaching the seas.

Projections of demographic growth coupled with increased demand and consumption of meat and dairy products prompted concerns for worsening of global ‘sanitation crisis’ (Vörösmarty et al., 2010), especially in rivers where discharge flows and dilution capacity are predicted to decrease as a consequence of climate changes. Wen et al. (2017) modelled BOD globally and concluded that, if wastewater treatment levels are kept at current level, the world population affected by organic pollution (BOD > 5 mg O_2_/L) will amount to 2.5 billion people by year 2050. For Europe, the study projected an increase in organic pollution especially in the Southern Countries due to loss of dilution capacity for rivers in water scarce areas. This projection partly contrasts with the European-wide assessment of Voß et al. (2012), which indicated that the Eastern part of Europe and the Black Sea were to be more affected by reduced dilution capacity of rivers and potential degradation of water quality.

Across Europe BOD is monitored systematically as an indicator of river water quality. An important decreasing trend in BOD concentrations has been detected in the last decade (EEA, 2015), largely thanks to the introduction of the Urban Waste Water Treatment Directive (EC, 1991), which has prompted investments in urban waste water treatments (EEA, 2015; Worrall et al., 2018). Legislative intervention has thus counter affected projected trends, however it is legitimate to question to which limit Urban Waste Water Treatment Plan (UWWTP) improvements may reduce BOD levels in European freshwater systems, and if this would be sufficient also to preserve or achieve good ecological status of freshwater systems as required in the Water Framework Directive (WFD; EC, 2000).

The aim of this study was to develop a pan European model to assess current BOD fluxes in freshwater systems. The conceptual model was inspired by previous large-scale models (Voß et al., 2012; Wen et al., 2017), but informed by detailed European databases for assessing current (2010s) BOD conditions. The model is called GREEN^+^_BOD_ because it shares the general conceptual structure and extent of the GREEN model (Grizzetti et al., 2012) to track fate of nutrient pollution in Europe. The model was built to assess mean annual BOD loads (t/y) and concentrations (mg O_2_/L) in freshwater reaches. It was calibrated against BOD concentrations monitored in Europe in 2008–2012. The model conceptualization addresses all potential sources of anthropogenic BOD, including BOD loadings from natural and urban areas, and adopts effective parameters that are suitable for the European conditions. Parameter sensitivity analysis, model calibration and validation are carried out to assess potential sources of uncertainty in the parameterization before presenting an assessment of current organic pollution across Europe. Finally current knowledge gaps and potential use of the model for management scenarios are discussed.

## 2 Materials and methods

### 2.1 The model structure

**Fig. 1:**
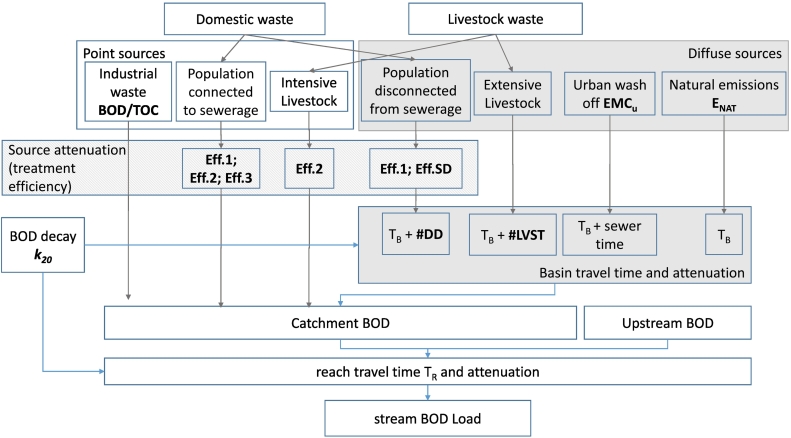
The structure of the model with BOD sources and attenuations scheme. Model parameters are highlighted in bold and defined in Table 1. Grey boxes indicate diffuse sources that are attenuated by basin retention before reaching the main stream network. The dashed grey box indicates attenuation at the source (treatment efficiency) applied before emissions to the environment.

The BOD model is built to assess mean annual 5-days BOD loads (t/y) and concentrations (mg O_2_/L). The spatial structure of the model is semi-distributed, based on subbasins that comprise a main reach and the land that drains into it. The main reaches are linked topologically from upstream to the river outlet. The extent and topological structure corresponds to the River and Catchment dataset for Europe (CCM2; Vogt et al., 2007), with subbasins of 7 km^2^ mean area (interquartile range 1–8 km^2^). Several sources of BOD are considered. Point sources of BOD loadings comprise urban waste water (UWWTPs discharges) and industrial discharges, and are considered to be discharged directly in the subbasin reach. Diffuse sources encompass organic waste from livestock, scattered dwellings, urban wash off, and organic matter originated from natural areas, like forests and inland waters. Abatement of BOD in waters was based on travel time. Diffuse sources are considered to be reduced in the subbasin before reaching the main stream network. Travel times from diffuse sources from basin release to the main reach are set separately for each source, allowing for different preferential pathways. BOD load in the stream network is degraded during transport as a function of the travel time in the reach (Fig. 1). Source conceptualizations and data are described in more detail here below.

#### 2.1.1 Domestic waste

Vigiak et al. (2018) describes how domestic waste emissions were assessed in detail. Briefly, all domestic waste generated from population that was connected to a sewer system was considered a point source discharged directly in the stream network. Conversely, disconnected population was considered a diffuse source of pollution and abated through basin retention before reaching the main stream network. Disconnected population was divided into scattered dwellings (SD), i.e. sparse homesteads that were assumed equipped with septic tanks, and domestic waste treated through Individual Appropriate Systems (IAS), which were assumed equivalent to primary treatment facilities.

The Waterbase-UWWTD database (EEA, 2017), which reports domestic waste generation, UWWTP treatment level, and location of discharges in 2014 was used as basis to assess IAS and UWWTP emissions from agglomerations larger than 2000 Person Equivalents (PE) in EU28 countries (listed in Supplementary material Table S1), Switzerland and Norway. Domestic waste for countries that are not covered in the database were estimated on the basis of population density and national statistics of connection to sewerage and treatment rates. Subbasin population was estimated from Global Human Settlement (GHS) population 1 km^2^ raster grid for 2015 (CIESIN, 2015). The percentage population connected to sewer system per treatment level was derived from national statistics for year 2015. Shares of population per treatment level were distributed spatially based on population density, assuming that most densely populated areas would benefit of the best treatments (Vigiak et al., 2018).

Comparison of Waterbase-UWWTD generated PE and population per country indicated an approximate rate of 1 PE to 1.23 inhabitants (R^2^ of linear regression = 0.98, sample size = 15; Vigiak et al., 2018). The 23% increase of PE compared to resident population can be interpreted as waste produced by commercial, industrial or touristic activities that is discharged in the sewerage systems. The PE/inhabitant rate was used to assess the amount of domestic waste generated in small agglomerations (<2000 PE), which is not reported in the UWWD database. GHS country population was compared to the equivalent population reported in the UWWTD database, i.e. the amount of generated PE divided by 1.23. When GHS population exceeded the reported population, the difference in population was considered as part of scattered dwellings or connected not treated population. Further, the PE/inhabitant rate was applied in countries unreported in the UWWD database to transform population into PE, thus aligning the two estimation methods. Sewerage losses applied to loadings generated by connected population were estimated at 10% (Morée et al., 2013).

BOD loadings from domestic waste were assessed considering 60 g BOD/PE/day as per UWWD database definition. Treatment efficiencies were assumed for septic tanks, primary, secondary and tertiary levels. Acknowledging uncertainty in efficiencies reported in literature (Nelson and Murray, 2008) and the range of technologies that can encompass each treatment level, treatment efficiencies were considered as model parameters within literature ranges (Table 1). After treatment abatement, point source emissions were attributed to the main reach of the subbasin that contained the WWTP discharge point coordinates or the agglomeration in case of connected not treated discharges. Conversely, emissions from disconnected population were subject to basin attenuation before being discharged in the subbasin main reach (Fig. 1) as in Grizzetti et al. (2012).

**Table 1:** Model parameter descriptions, initial ranges based on literature, and calibrated values.

Parameter	Description	Initial range	Calibrated
Eff.1 [0–1]	BOD efficiency primary treatment	0.35–0.75	0.50
Eff.2 [0–1]	BOD efficiency secondary treatment – applied also to high density livestock treatment	0.8–0.95	0.94
Eff.3 [0–1]	BOD efficiency tertiary treatment	0.92–0.98	0.96
Eff.SD	BOD efficiency septic tank	0.35–0.40	0.40
#DD [days]	Retention days in excess of basin travel time employed by disconnected domestic waste to reach stream network	4–8	7
#LVST [days]	Retention days in excess of basin travel time employed by low density livestock waste to reach stream network	4–8	7
*k*_20_ [days^−1^]	Freshwater BOD retention	0.35–0.60	0.56
EMC_U_ [mg O_2_ L^−1^]	Effective mean concentration of BOD in combined sewer overflows in urban areas	1–21	11
E_NAT_ [t km^−2^ y^−1^]	BOD export coefficient of natural areas	0–0.5	0.16
bod/toc	Ratio BOD/TOC in industrial waste	0.2–2.2	0.75

#### 2.1.2 Industrial emissions

Emissions from industries connected to UWWTPS were accounted for as part of domestic waste. Additionally, industrial releases from large facilities that directly treat and discharge their waste were taken from the European Pollutant Release and Transfer Register database (E-PRTR; EEA, 2018). The database reports annual emissions for facilities of European Union members, Iceland, Liechtenstein, Norway, Serbia and Switzerland from 2007 to 2016. UWWTPs included in this database were removed to avoid duplications with domestic releases. Annual facility releases to water (net to transfers) of Total Organic Carbon (TOC) for the seven-year period 2010–2016 were averaged to obtain mean annual facility TOC emissions (t/y). The ratio BOD/TOC in industrial effluents depends on the type of industrial waste and treatment applied, and may range from as low as 0.1–0.6 in petrochemical effluents, to 0.5–0.75 in pulp and paper mill facilities, to >2 for agro-industries (Dubber and Gray, 2010; Mook et al., 2012; Quayle et al., 2009; Banerjee and Ghoshal, 2017; Christian et al., 2017; Bustillo-Lecompte et al., 2018; Grötzner et al., 2018; Kwarciak-Kozłowska and Bień, 2018). Thus, the BOD/TOC ratio was introduced as a model calibration parameter to assess BOD sources from industries (BOD = TOC*bod/toc; Table 1). Releases were attributed to the subbasin that contained the facility spatial coordinates, and considered point sources, i.e. directly discharged to the main reach (Fig. 1).

#### 2.1.3 Livestock waste

Livestock waste is a major source of BOD pollution globally (Wen et al., 2017, Wen et al., 2018) and in Europe (Malve et al., 2012). The 1 km^2^ global distribution of livestock maps for the reference year 2005 (Robinson et al., 2014) were used to assess livestock heads per type and subbasin. BOD from livestock waste was estimated following Wen et al. (2017). Briefly, the number of animals was transformed in Livestock Units (LU), which produce 400 g BOD/LU per day. Livestock waste was divided into intensive or extensive production systems (Fig. 1). Intensive systems comprised all pig and chicken LUs, as well as cattle and goat/sheep at density higher than 25 LU/km^2^. Intensive systems were considered of industrial type and BOD emissions were treated at secondary level before being discharged to the main reach as point sources. Conversely, extensive systems comprised cattle and goat/sheep bred at density lower than 25 LU/km^2^. BOD emissions from extensive systems were considered diffuse in the catchment, and abated through basin retention before reaching the stream network as in Wen et al. (2017).

#### 2.1.4 Urban wash off

BOD washed off by urban runoff is mostly collected in sewerage systems and treated by UWWTPs. However, during intense storms sewers may get overloaded, and the excess runoff is spilled to the environment. To account for these occurrences, we considered urban land as an additional source of diffuse BOD not accounted for through domestic waste. Urban BOD was estimated as proportional to annual urban runoff volume (m^3^) occurring in a subbasin:

(1) $${\mathrm{BOD}}_{U}={\mathrm{EMC}}_{U}*R_{U}*3600*24*365.25/1000000$$

where BOD_U_ = urban wash off BOD (t/y); R_U_ = annual urban runoff (m^3^/s); and EMC_U_ is the effective mean annual BOD concentration in urban wash off (mg O_2_/L; Table 1). Williams et al. (2012) set EMC_U_ at 11 mg O_2_/L but then considered it to be treated and abated in UWWTPs. Andrés-Doménech et al. (2018) reported BOD event mean concentrations of 5–22 mg O_2_/L for Valencia in South Spain. Literature reports large ranges in BOD effective mean concentrations from urban nonpoint sources (Rossman and Huber, 2016; Hur et al., 2018). Given the paucity of data in amount of urban wash off occurrences and BOD mean concentrations, we set a large initial range of this parameter (Table 1).

#### 2.1.5 Natural areas emissions

Organic matter washed off from natural areas to freshwater systems contributes to biodegradable material and to BOD monitored in running waters (e.g. HELCOM, 2004). Soils in natural areas are richer in litter and organic matter content than agricultural fields (Aitkenhead and McDowell, 2000; Smagin et al., 2018) and may contribute to organic matter in freshwater systems significantly (Povilaitis, 2008; Borrelli et al., 2018a, Borrelli et al., 2018b). However, the relationship between organic matter originated from natural areas and BOD in streams is not straightforward as biodegradability depends on the type of organic material (Aitkenhead and McDowell, 2000; HELCOM, 2004; Kwak et al., 2013; Liu et al., 2015).

Additionally, lakes (including artificial reservoirs) can act as sinks or sources of BOD to downstream waters. Their role depends on several factors, among which trophic status, hydrology and retention time, type and amount of incoming organic matter, and location within the freshwater network (Epstein et al., 2013; Tipping et al., 2016; Hastie et al., 2017). Autochthonous releases of organic matter are generally more biodegradable than incoming sources, for examples when particulate organic matter in sediments is transformed into more liable dissolved organic components (Epstein et al., 2013; Cunha-Santino et al., 2013; Ejarque et al., 2018). As a result, BOD outflowing flux may be higher than BOD influx (Cunha-Santino et al., 2013). Incoming organic matter fluxes, trophic status, and hydrology vary with seasonality, thus the same lake can switch from source to sink of carbon during rainfall events and seasons (Goodman et al., 2011; Ejarque et al., 2018). Specific assessment of BOD fluxes in lakes was out of the scope of this work, however lakes were included as part of natural emissions of BOD to downstream waters.

Emissions of BOD due to organic material either washed off from natural areas or released from lakes were pooled together and assumed to be proportional to the extent of natural land *Area*_*NAT*_ (km^2^). Natural areas were defined as subbasin area not covered by agriculture or urban land according to CORINE Land Cover 2012 (CLC, 2012). A simple export coefficient method with a constant area emission factor (E_NAT_; t/km^2^) was adopted:

(2) $${\mathrm{BOD}}_{\mathrm{NAT}}=E_{\mathrm{NAT}}{\mathrm{Area}}_{\mathrm{NAT}}$$

Povilaitis (2008) estimated BOD contribution from forest and grassland in a Lithuanian catchment to be around 0.5 t/km^2^; Shrestha et al. (2008) estimated a forest export coefficient of 0.1 t/km^2^. Aitkenhead and McDowell (2000) estimated dissolved organic fluxes from biomes to vary from 1 kg/ha in cool grasslands to about 100 kg/ha from peats. A large variability of emission could be expected not only in consideration of amount and type of organic matter in the soil, but also on net erosion rates (Borrelli et al., 2018a, Borrelli et al., 2018b), and enrichment ratio in washed off particles as well as rate of autochthonous releases from lakes. To account for large variability of this BOD source, a relatively large initial range of the export coefficient E_NAT_ was set in model calibration (0–0.5 t/km^2^; Table 1).

#### 2.1.6 BOD decay in freshwaters

All sources of BOD were estimated as mean annual BOD load (t/y). Before reaching the main stream network, diffuse BOD sources are however attenuated by degradation occurring during the time necessary to arrive to the main reach and in transport through the main freshwater network. BOD attenuation in freshwater was modelled with an exponential decay as a function of travel time (Wen et al., 2017), both during the transport within the subbasin to the stream network and in the main subbasin reach. BOD load flux was thus assessed as:

(3) $${\mathrm{BOD}}_{\mathrm{OUT},j}={\mathrm{BOD}}_{\mathrm{IN},j}e^{-k_{T,j}{\mathrm{TT}}_{j}}$$

where *j* refers to the *j*th subbasin or reach; BOD_IN_ = BOD influx (t/y), and TTj is the mean travel time in the subbasin (TT_B_) or reach (TT_R_; days); *k*_*T,j*_ is the BOD decay in the *j*th subbasin; and BOD_OUT_ = BOD outflux (t/y). As in Wen et al. (2017), the first order degradation rate coefficient *k*_*T,j*_ was derived from the decay rate at 20 °C k_20_ and adjusted with Arrhenius equation to account for water temperature:

(4) $$k_{T,j}=k_{20}1.047^{\left(\mathrm{Tj}-20\right)}$$

where *k*_*T,j*_ is the degradation rate [/day] in the *j*th subbasin, *k*_20_ is the degradation rate at 20 °C and T_*j*_ is the water temperature [°C] in the subbasin. Water temperature across Europe was assessed with a log-linear function of mean annual air temperature (Vigiak et al., 2017; GLOBAQUA, 2018).

Wen et al. (2017) adopted a value of *k*_20_ of 0.35/day, whereas Voß et al. (2012) had set it to 0.23/day. Large variability in *k*_20_ has been measured in laboratory (0.3–0.5/day) or in rivers (Catalán et al., 2016; Higashino and Stefan, 2017). Rather than setting it a priori, *k*_20_ was included in the calibration parameter set (Table 1).

#### 2.1.7 Subbasin travel time (*TT*_*B*_)

Travel time in the subbasin was based on time lag estimated as a simple empirical function of subbasin area (Mimikou, 1984; Gericke and Smithers, 2014):

(5) $$T_{B}=0.43{A_{B}}^{0.418}$$

where *T*_*B*_ = subbasin time lag (h), and A_B_ = area of the subbasin (km^2^); *T*_*B*_ was transformed in days for application in Eq. (3). The relative small size of CCM2 subbasins resulted in time lag mostly lower than 0.1 days (mode of 0.003 days, median of 0.03 days), up to 0.4 days in large subbasins.

*T*_*B*_ is an approximation of travel time in the subbasin via surface flow. However, diffuse sources may follow other pathways, e.g. via interflow, with longer travel time. To adapt travel time by different pathways, source specific delays [days] were added to *T*_*B*_ to obtain the source travel time in the subbasin. Domestic waste from disconnected dwellings (SD and IAS) was assumed to leach in the underground and reach the stream network through groundwater or interflow. To account for delay through this pathway an additional time (#DD; in days) was added to the time lag (*TT*_B,DD_ = *T*_*B*_ + #DD; Fig. 1, Table 1). Extensive livestock waste is supposed to mix in the soil or stay on the soil after application for some time before being washed off to the stream network. This delay (#LVST; days) was kept distinct from that of domestic waste and included in the model as a separate calibration parameter (*TT*_B,LVST_ = *T*_*B*_ + #LVST; Fig. 1, Table 1). Finally, urban runoff collected in sewerage systems was delayed by time spent in sewers (*TT*_B,U_ = *T*_*B*_ + sewer time), assumed equal to 4 h (Ort et al., 2014; Kapo et al., 2017). Conversely, no additional delay was applied to natural area emissions, as organic matter is transported to stream network mainly by surface flow, thus in this case basin travel time was assumed equal to subbasin time lag (*TT*_B,NAT_ = *T*_*B*_).

#### 2.1.8 Reach travel time (*TT*_*R*_)

Travel time in the reaches of the main streamflow network *TT*_*R*_ (days) was calculated on the basis of reach length and water velocity. Water velocity was estimated from mean annual discharge, assuming hydraulic relationships suitable for the European continent (Pistocchi and Pennington, 2006). Mean annual discharge was taken as the mean flow simulated for 2005–2013 with the calibrated pan European LISFLOOD model (Burek et al., 2013). The median stream travel time in reaches *TT*_*R*_ was 0.03 days; in 50% of reaches it ranged from 0.01 to 0.07 days, and was up to 3.75 days in the longest reaches.

### 2.2 BOD monitored dataset

BOD monitored in rivers across Europe was retrieved from Waterbase Rivers (EEA, 2016). Since BOD has been declining after 2007 (EEA, 2015), only the last five years (2008–2012) of available data were used for this study. The database reports mean annual or seasonal BOD5 or BOD7 concentrations (mg O_2_/L), and sample size. Only mean annual values of sample sizes larger than six measurements/year were used in this study as smaller sample size was considered unreliable. After taking out data entries flagged in the database as outliers or as failing quality assurance checks, 11,115 data in 3252 stations were retained for model calibration and validation. The modal reported detection limit of BOD in the retained database was 0.5 mg O_2_/L, and about 50% of data entries had detection limits below 1 mg O_2_/L. Furthermore, BOD7 loads (t/y) for 29 stations in 2008–2015 were provided by HELCOM (2017). BOD7 data were transformed in BOD5 as BOD7/1.16 (EEA, 2016).

**Fig. 2:**
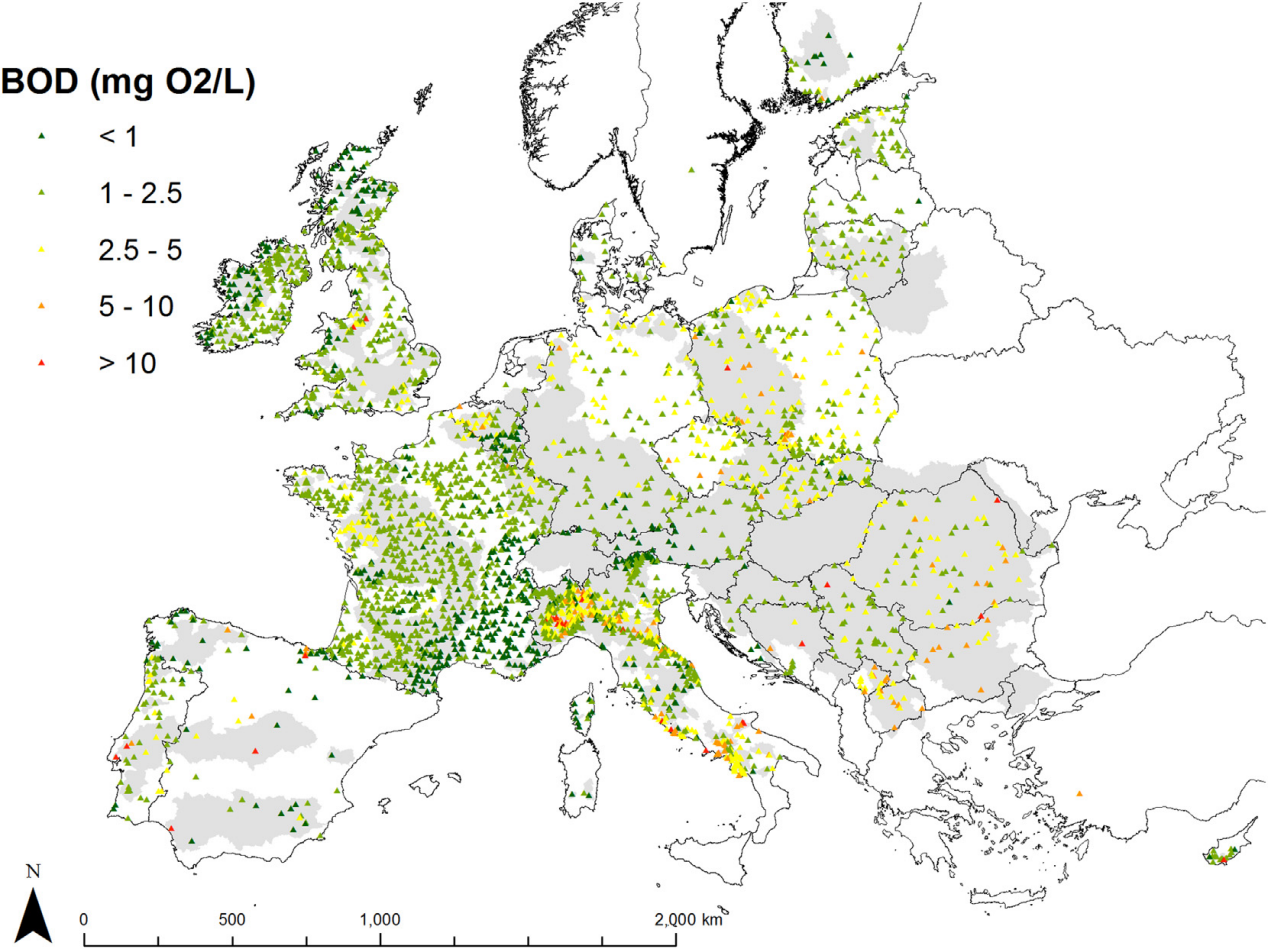
Mean annual BOD concentration (mg O_2_/L) monitored data used in this study. Grey background show basins whose data was used for model calibration.

**Fig. 3:**
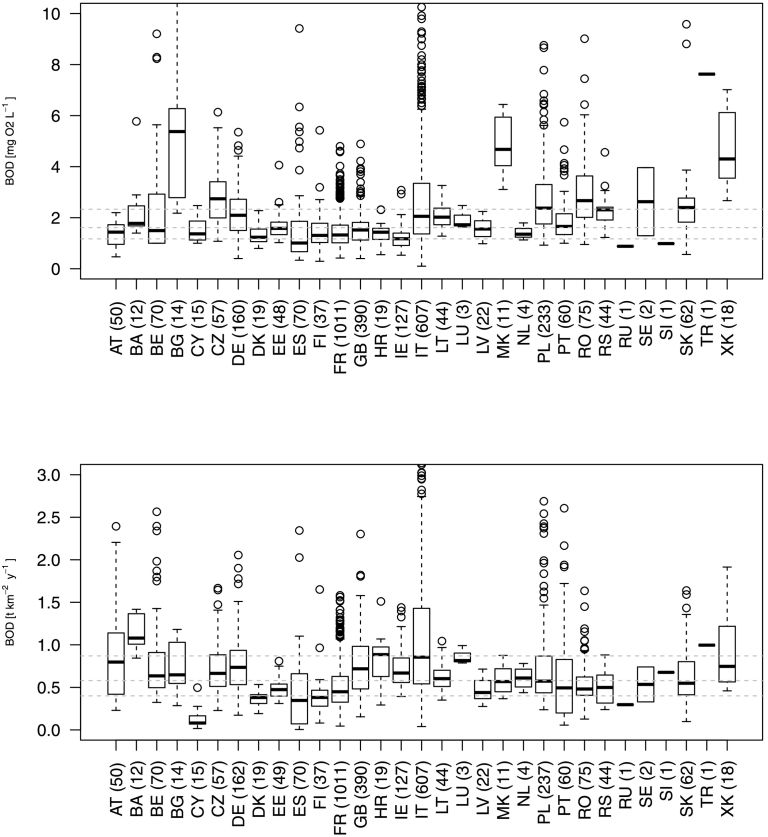
European dataset of mean annual BOD monitored concentrations (mg O_2_/L; above) and loads (herein reported as specific loads, t/km^2^/y; below). Number in brackets indicate the number of stations per country. Country codes are defined in Supplementary material S1.

Mean BOD concentrations per station from EEA (2016) were transformed in loads using a long-term mean annual streamflow. Similarly, HELCOM load data were transformed into mean annual concentrations, except in two cases for which streamflow was very small, and probably erroneous, and transformation into concentration would create large outliers. In total, 3287 mean annual BOD concentrations and 3291 loads could be used for model. Monitored BOD in the dataset ranged from 0.1 to 41.2 mg O_2_/L with median of 2.11 and interquartile range 1.17–2.33 mg O_2_/L. Importantly, the spatial distribution of monitored data varied largely across countries, with the densest set located in France but no data from Greece or Hungary (Fig. 2). A considerable variation in BOD monitored data could be observed among countries, for example, BOD concentrations appeared high in Kosovo,^1^ FYR Macedonia and Bulgaria, whereas BOD specific loads (t/km^2^/y) were higher than mean European values in Bosnia and Herzegovina and lower in Cyprus (Fig. 3).

### 2.3 Model calibration and validation

The model requires ten parameters (Table 1), for which initial plausible ranges could be well defined based on literature. Model calibration was conducted on a subset of monitored dataset: 218 European basins selected from different European regions and with size ranging from 35 to 802,000 km^2^ (geometric mean size 1275 km^2^), on which 2157 (65%) BOD monitoring stations fell (Fig. 2). Validation was conducted on the remaining basins, with 35% data (1134 stations). Calibration was made adopting uniform distribution of parameters within the established ranges, and sampled via a Latin Hypercube scheme to generate 1500 parameter sets. As BOD data were not normally distributed, but showed a prevalence of low values, BOD concentrations were log-transformed to evaluate fit of the 1500 model simulations. A further evaluation of model results was conducted on an independent dataset available in four European basins (see Supplementary material).

## 3 Results

### 3.1 Model calibration and validation

**Fig. 4:**
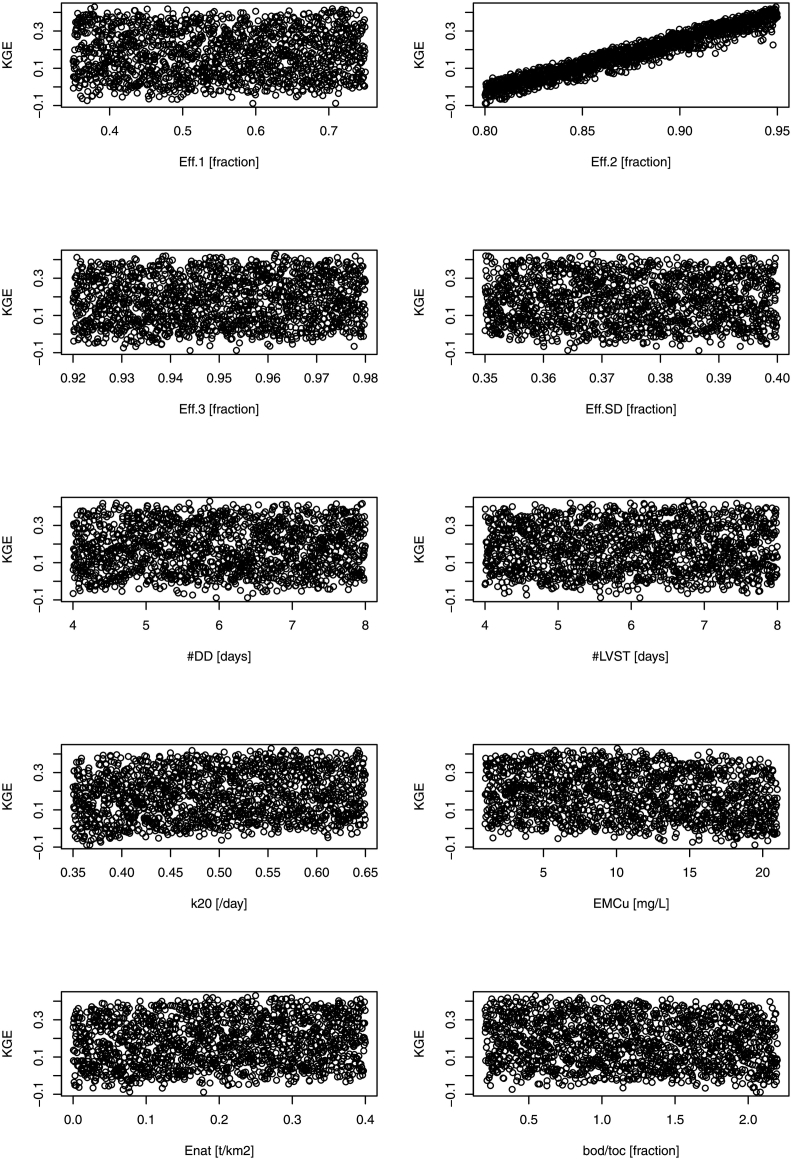
Kling-Gupta efficiency (KGE) of 1500 model runs using uniform distribution within the calibration ranges. Parameters are described in Table 1.

**Fig. 5:**
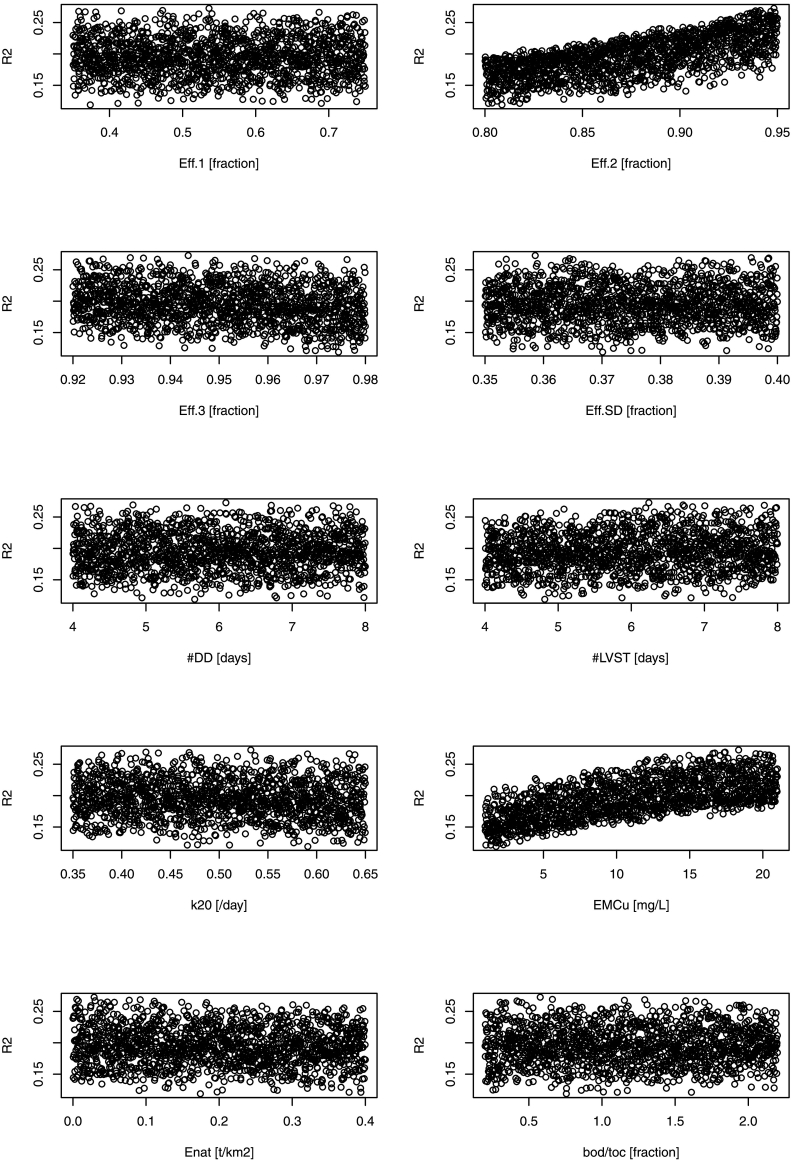
Coefficient of determination (R^2^) of 1500 model runs using uniform distribution within the calibration ranges. Parameters are described in Table 1.

Dot plots of simulations were analyzed to understand parameter sensitivities. Among several goodness of fit indices, two were found to respond differently to parameters, the Kling-Gupta efficiency (KGE; Gupta et al., 2009; Kling et al., 2012), which is a measure of model efficiency but weighted for correlation and variability of dataset, and the coefficient of determination R^2^. KGE was highly responsive to the efficiency of secondary treatments (Eff.2; Fig. 4), improving sharply with increasing Eff.2, whereas response of other parameters was unclear. The R^2^ was also responsive to Eff.2, but was also sensitive to parameters regulating urban and natural area emissions, EMC_u_ and E_NAT_ (Fig. 5).

Within the 1500 simulations, we identified a subset of best parameter sets (‘behavioral’ using the terminology of Beven and Freer, 2001), i.e. parameter sets that would best fit the observations while remaining in an acceptable physical ranges, as those that scored in the upper quartile of KGE and R^2^. This selection yielded 102 parameter sets. Within the behavioral parameters subset, some linear correlations between model parameters could be identified (Table 2; the distribution of model parameters in the behavioral dataset is in Supplementary material). KGE increased with efficiency of secondary treatment Eff.2, BOD decay *k*_20_, and natural emissions (E_NAT_), and with decreasing urban wash-off (EMC_U_). R^2^ increased with Eff.2, livestock travel time delay (#LVST), and EMC_U_, but decreased with E_NAT_. Thus model response to KGE and R2 was opposite for EMC_U_ and E_NAT._ Few significant correlations were found among model parameters: Eff.1 was positively correlated to industrial bod/toc ratio; effective concentration in urban wash-off (EMC_U_) was negatively correlated to efficiency in secondary treatment but positively correlated to that of tertiary treatment. Efficiency in septic tanks (Eff.SD) was correlated to efficiencies in secondary or tertiary treatment but was not correlated to model goodness-of fit indices.

**Table 2:** Pearson's correlation matrix of model parameters and with goodness-of-fit measures KGE and R^2^. Values in bold indicate significant correlation at *P* < 0.05.

Parameter	Eff.1	Eff.2	Eff.3	Eff.SD	#DD	#LVST	*k*_20_	EMC_U_	E_NAT_	bod/toc	KGE	R^2^
Eff.1	1											
Eff.2	−0.10	1										
Eff.3	−0.03	−0.01	1									
Eff.SD	0.10	**0.22**	**−0.21**	1								
#DD	−0.02	−0.11	0.03	−0.09	1							
#LVST	−0.09	−0.11	0.08	−0.05	0.03	1						
*k*_20_	−0.06	−0.11	0.01	−0.05	−0.10	−0.15	1					
EMC_U_	0.10	**−0.23**	**0.26**	−0.09	0.04	−0.17	0.15	1				
E_NAT_	−0.02	0.01	−0.10	−0.01	0.13	0.05	0.02	0.08	1			
bod/toc	**0.23**	−0.01	0.06	0.06	−0.06	−0.02	0.00	0.09	0.06	1		
KGE	−0.17	**0.68**	0.12	0.07	0.15	0.09	**0.26**	**−0.48**	**0.22**	**−0.25**	1	
R^2^	−0.05	**0.46**	−0.16	0.15	0.05	**0.26**	−0.15	**0.30**	**−0.36**	0.00	0.03	1

**Fig. 6:**
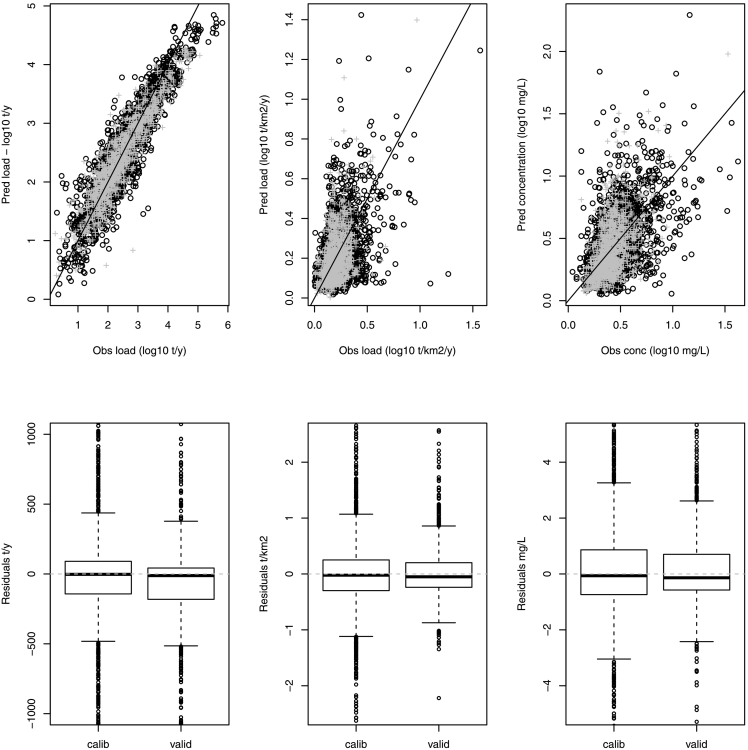
Above: modelled (y axis) vs observed (x axis) BOD fluxes (in logarithmic scale), from left to right: mean annual load (t/y); specific loads (t/km^2^/y); and concentrations (mg O_2_/L). Calibration dataset is indicated with black circles and validation dataset in grey crosses. Below: boxplots of modelled flux residuals (normal scale) for calibration and validation datasets.

Using these information, a final calibration parameter set was identified (Table 1), which resulted in mean predicted BOD concentrations for all monitored stations of 2.54 mg O_2_/L (compared to 2.10 mg O_2_/L of observations); the 90th percentile of predicted BOD concentration was 4.76 mg O_2_/L, while that of monitored data was 3.51 mg O_2_/L. In the calibration dataset, the percent bias of BOD concentration was 20% and that of BOD specific loads was 1% (Table 3). Model percent bias slightly worsened in the validation dataset, which would be expected in split calibration-validation modelling (Fig. 2). Scatter plots of predicted versus observed BOD fluxes and boxplots of model residuals (Fig. 6) shows the presence of some large errors in predicting specific loads or concentrations. Yet, model residuals were centered on zero and there was no statistical indication of a bias of model toward over or under predictions. In consideration of these results, the model was considered fit to predict water quality for organic pollution across Europe.

**Table 3:** Summary statistics of measured BOD and mean error statistics of BOD model predictions (concentrations and specific loads) in the calibration and validation datasets. Model parameters are in Table 1. MAE = mean absolute error. RMSE = root mean Square Error.

		Calibration		Validation	
		Concentration mg O_2_/L	Load t/km^2^	Concentration mg O_2_/L	Load t/km^2^
Observations	Sample size	2156	2157	1131	1134
	5th percentile	0.73	0.21	0.66	0.22
	Mean	2.29	0.81	1.75	0.62
	95th percentile	5.65	1.92	3.62	1.21
	Max	41.18	36.14	32.8	8.34
Prediction errors	MAE	1.72	0.52	1.21	0.37
	RMSE	5.47	1.20	3.04	0.79
	Percent bias %	19.6	0.6	24.2	8.9

**Fig. 7:**
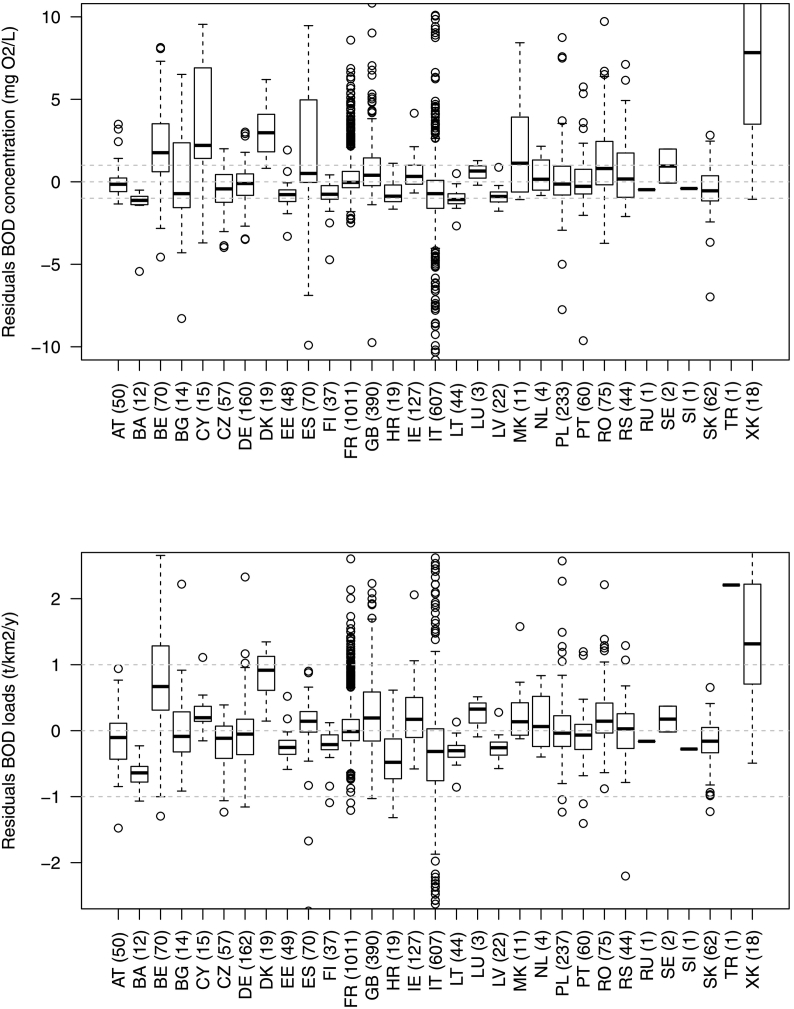
Model residuals (predicted-observed) BOD fluxes (concentrations and specific loads) per country. Number in brackets indicate the number of stations per country. Dashed grey horizontal lines indicate null and ±1 errors. Country codes are defined in Supplementary material S1.

**Fig. 8:**
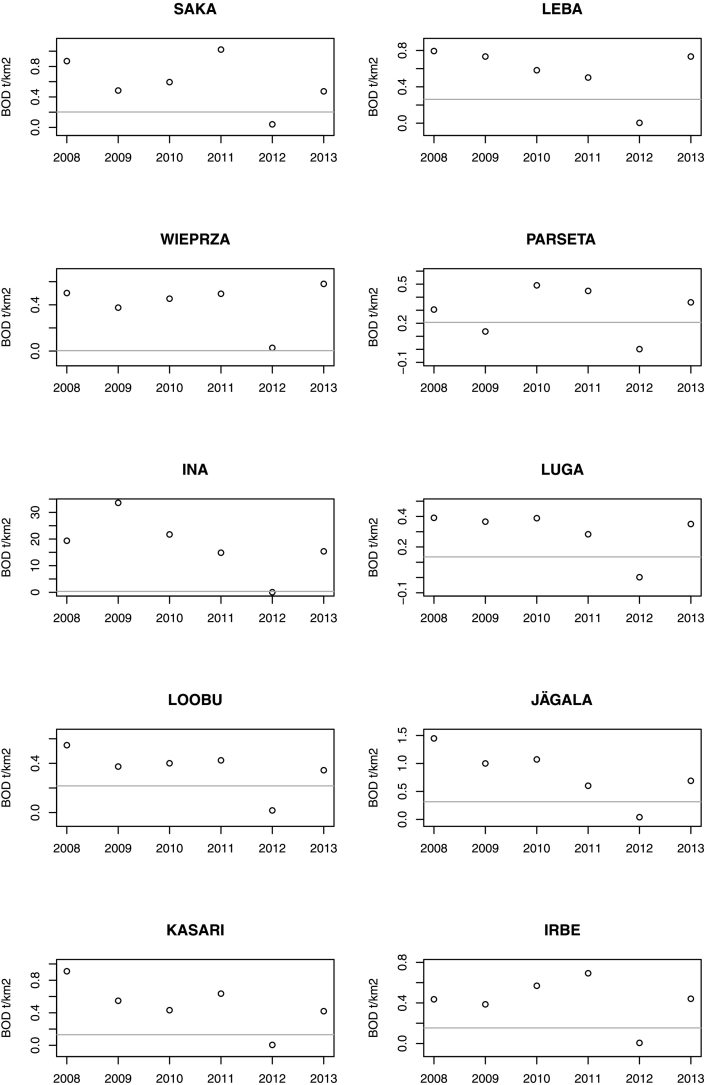
BOD modelled specific load (continuous grey line) and annual observed loads for ten stations for which six years of BOD load observations were available (HELCOM dataset).

In terms of geographical distribution of model errors (Fig. 7), a prevalence of overestimations in some countries, e.g. Belgium, Denmark, Cyprus, FYR Macedonia, and Kosovo could be noted. Conversely, a prevalence of model under predictions occurred in Bosnia and Herzegovina, Croatia, Italy, and Slovakia. No appreciable bias could be detected in other countries, like Germany, France, Poland and Serbia. Fig. 8 shows model prediction (BOD specific loads) for the stations with the longest monitoring period (6 years; all part of HELCOM dataset).

Classes of water quality for organic pollution can be expressed in terms of BOD concentration thresholds. While there are no unified guidelines for the European continent, high water quality is considered when BOD concentrations are below 2–3 mg O_2_/L, good quality below 4–5, moderate below 7–10; poor if concentrations are above 10, and bad when it exceeds 15–25 mg O_2_/L (Kristensen and Hansen, 1994; Brunel et al., 1997; IT, 1999; Wen et al., 2017; Mladenović-Ranisavljević et al., 2018). Considering these general guidelines, a confusion matrix between model predictions and observations per water quality class was derived (Table 4). The overall accuracy of the model was 68.6%. Allowing for an error of 0.5 mg O_2_/L at the thresholds, the agreement between modelled and observed concentration raised to 73.7%. However, the model appeared to under predict poor BOD areas, and accuracy degraded from high to bad quality classes. Errors of over-predictions amounted to 1.6% whereas under-predictions were 4.7%.

**Table 4:** Confusion matrix of modelled and observed BOD concentrations (all European monitored dataset) divided in classes of water quality based on European and global guidelines.

Predicted BOD class	Observed BOD class (mg O_2_/L)						User's accuracy
	High	Good	Moderate	Poor	Bad	Total	
	< 2.5	2.5–5	5–10	10–15	> 15		%
High	2027	432	98	11	13	2581	78.5
Good	257	186	79	12	12	546	34.1
Moderate	32	31	37	18	8	126	29.4
Poor	3	8	5	3	3	22	13.6
Bad	0	3	5	2	2	12	16.7
Total	2319	660	224	55	38	3287	
Producer accuracy %	87.4	28.1	16.5	5.4	5.3		68.6

### 3.2 BOD fluxes in Europe

**Fig. 9:**
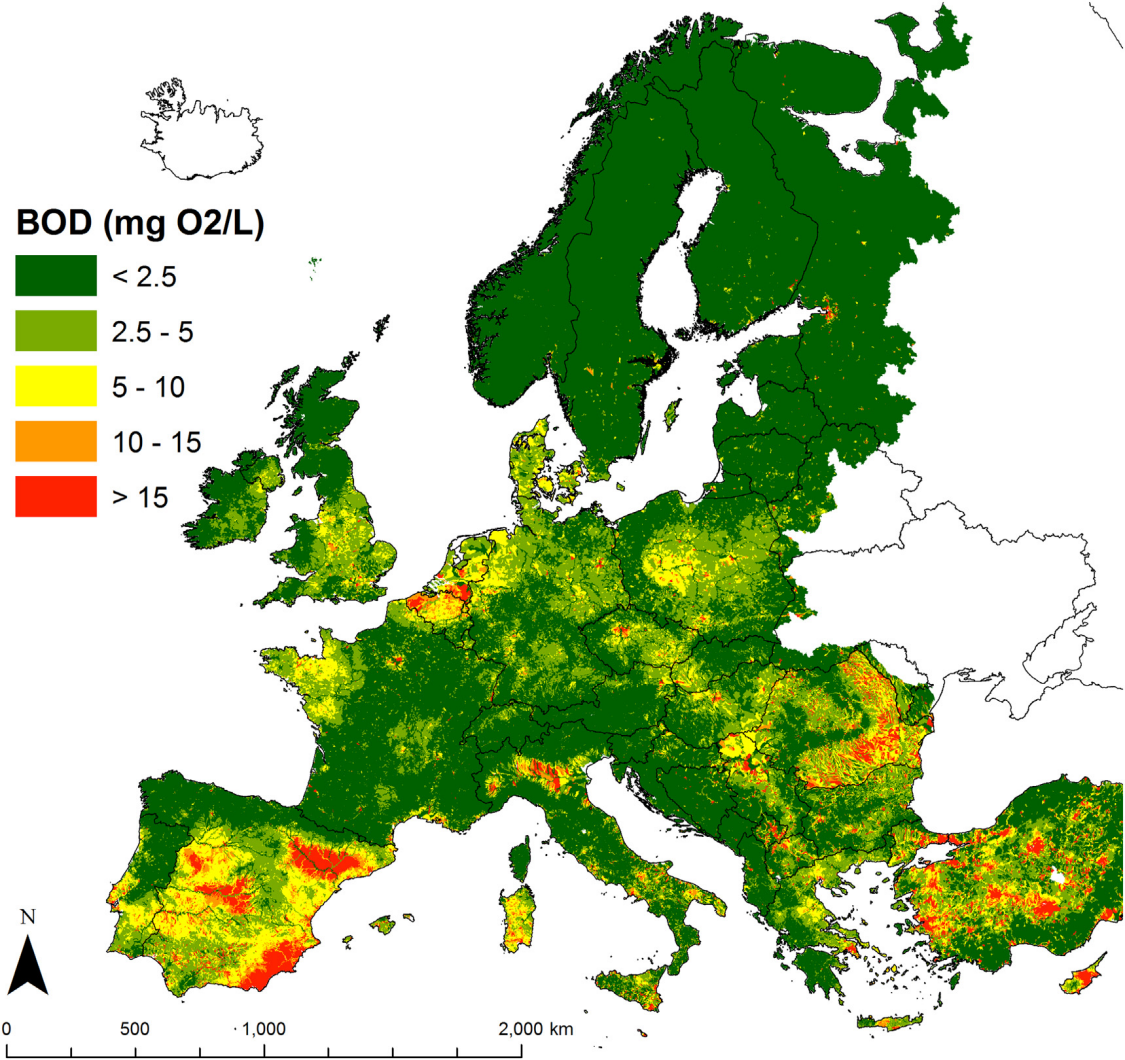
Map of BOD concentration (mg O_2_/L) in European freshwaters predicted for the 2010s.

BOD concentrations were mapped in Europe adopting the same class thresholds of Table 4 (Fig. 9). While about two thirds of freshwater network were estimated as being in high conditions (mean annual BOD < 2.5 mg O_2_/L), about 12% (262,000 km) of freshwater network for the whole territory, (14%, or 210,000 km, in the EU28 zone) was mapped to be failing good quality due to excessive BOD concentrations (>5 mg O_2_/L). Areas of high BOD concentrations were linked to high urban density, especially where untreated domestic waste is still sizable, in zones of high livestock densities, and/or where dilution is limited by low streamflow, like in the Mediterranean region.

BOD loadings discharged to the sea were estimated at 2.4 M t/y annually (Table 5). Anthropogenic sources were domestic waste (725 kt/y; 30%), livestock (639 kt/y, 27%), industry emissions (474 kt/y; 20%), and urban wash off (336 kt/y; 14%). Of livestock waste, 78% (501 kt/y) originated from intensive production systems. Natural areas emissions contributed 229 kt/y (10%) of loadings to the seas (Table 5). Notably, coastal catchments, i.e. catchments that quickly discharged to seas and identified as having Shreve (1966) order of 1 in CCM2 topology, which occupies only about 4% of land, contributed 869 kt/y (36%) of the BOD loadings to the seas, mostly (77%) from point source emissions either industrial (46%) or domestic (31%). In North Europe, livestock emissions, estimated to contribute 24–53% of BOD loadings to the sea, and were the major source of BOD, and were followed by industrial emissions (15–72%). Domestic waste was a dominant source of BOD to the Mediterranean Sea and to the Black sea. Unsurprisingly considering the extent of forest land and lakes, natural area emissions were relatively more important in the Scandinavian countries and to Baltic Sea. Large variability in BOD sources could also be observed among river basins (Table 6).

**Table 5:** BOD loadings (t/y) to European Marine regions and major BOD sources estimated for the 2010s.

Marine region	BOD loading	Sources contribution to the total BOD loading to the sea					Coastal areas^a^ contribution to the total BOD loading
		Domestic	Industrial	Livestock	Urban wash off	Natural areas	
	t/y	%	%	%	%	%	%
Greater North Sea, incl. the Kattegat and the English Channel	598,911	18	22	37	18	5	33
North East Atlantic Ocean	301,117	5	72	2	1	19	80
Baltic Sea	284,739	21	19	24	15	22	28
Celtic Seas	236,270	14	15	53	15	4	34
Aegean-Levantine Sea	203,625	66	0	16	9	8	30
Bay of Biscay and the Iberian Coast	187,796	26	9	37	21	7	26
Western Mediterranean Sea	171,146	44	3	21	23	9	31
Black Sea	151,217	69	1	18	7	6	18
Adriatic Sea	135,321	41	8	26	19	7	25
Black Sea – sea of Marmara	92,366	81	0	8	8	2	37
Ionian Sea and the Central Mediterranean Sea	40,071	41	3	23	22	12	25
Total	2,402,580	30	20	27	14	10	36

a Coastal areas are defined as catchments that quickly discharge to the sea, i.e. Shreve order of 1.

**Table 6:** BOD loadings (M t/y) to European seas and major BOD sources (%) estimated for the 2010s in this study for the European river with the highest BOD load to the sea.

River	BOD loading	Domestic	Industrial	Livestock	Urban wash off	Natural areas
	t/y	%	%	%	%	%
Danube	33,740	67	1	17	11	4
Meuse	31,918	14	3	65	16	2
Rhine	29,803	33	9	21	34	4
Po	26,888	13	28	42	15	3
Escout/Schelde	24,910	32	3	35	30	1
Neva	23,406	52	11	7	1	29
Elbe	18,140	23	6	39	28	4
Thames	17,233	67	0	6	26	1
Loire	17,096	11	2	63	21	4
Wisla	15,806	21	10	45	18	7

## 4 Discussion

The conceptual model developed in this study builds on literature research (Voß et al., 2012; Wen et al., 2017), but expanding the roles of urban and natural areas as sources of BOD to freshwaters. As all conceptual models (e.g. Grizzetti et al., 2012), the model aims at assessing the importance of dominant sources of pollution and at tracking the fate of pollutant in the environment through simplifications that are dictated by an incomplete knowledge of processes and ultimately driven by calibration. Several sources of error may contribute to the scatter in model predictions (Fig. 6), related to errors or incompleteness of input and observation data, as well as to the model structure and the calibration procedure. Errors in model estimation ultimately depend on the interactions between all these sources of uncertainty. Here below we try to analyze major sources of errors and limits of the model.

### 4.1 Model prediction errors

Errors in location of monitoring stations, and by consequence attribution of draining area, can cause considerable deviation in deriving specific loads from the modelled outputs, i.e. mean annual loads. In consideration of the size of spatial units, which are relatively small, this error is likely to play an important role in the evaluation of model results. Location errors would also affect the attribution of mean annual streamflow which is used to transform loads into concentrations and vice versa. Where ancillary information about monitoring stations, like drainage area and streamflow, were reported, these data were used to countercheck location of monitoring stations. However this was rarely possible and location errors could not be excluded.

Monitored BOD data is subject to large uncertainty. First of all, BOD measurements in rivers are subject to a relatively large error, about 15–28% (Kwak et al., 2013). Plus, sampling frequency and averaging techniques impact mean annual records in datasets (Chappell et al., 2017). Discrepancies in reported BOD from different sources may be due to different sampling frequency (e.g. Supplementary material S2). Temporal variability and frequency of monitoring also affect mean annual values per station. In Fig. 8 some examples of the temporal variability in BOD loads can be appreciated, e.g. in the drop for all 2012 entries.

Errors in the estimation of mean annual streamflow may play an important role. The Waterbase Rivers database does not report streamflow associated to the water quality data entries, which represents a large limitation in the usefulness of these data for model evaluation, as the impact of streamflow on concentration cannot be quantified. In the model, we relied on long-term mean annual streamflow to derive modelled concentrations (and loads from monitored concentrations), and inter annual streamflow variation could not be taken into account. Further, water abstractions may not be fully considered, thus mean annual streamflow might be overestimated where abstractions are high. For example, in Cyprus BOD concentrations were generally overestimated in the model, whereas specific loads were not (Fig. 7). It may be that in this water scarce country, abstractions are not fully accounted for and mean annual streamflow is over predicted, leading to under predict BOD concentrations.

### 4.2 Uncertainty in model inputs

Missing model inputs and errors of input location, for example of BOD point source emissions, may also contribute to large local errors. For example, while BOD specific loads in eight out of the ten stations depicted in Fig. 8 were generally well predicted, the very high specific load of station INA (and to a lesser extent WIEPRZA) was completely underestimated by the model. In the model set-up, the station is located in small catchment (85.7 km^2^) in which there is no point source emission. It is unlikely that such high BOD specific loads could be generated in the absence of point sources, thus this example points to either an error in the location of monitoring station or a missing BOD point source.

In terms of uncertainty in input data, domestic waste was assessed to generate 2.3 M t/y, 70% of which discharged as point sources. In EU28, Switzerland and Norway domestic waste was based on the Waterbase-UWWTD database. The database provides unprecedented detailed information of population equivalent generation and discharge. While reporting inconsistencies in the database were noticed and dealt with (Vigiak et al., 2018), the main source of uncertainty of this input data source resides likely in the location of discharges, as errors in spatial coordinates may cause misallocations of point sources. For the other countries, domestic waste was estimated based on population density and national statistics. In this case, uncertainty in data input is mostly dependent on the reliability of national statistics, which for several countries is erratic (Williams et al., 2012; Sato et al., 2013). A second source of uncertainty is the spatial attribution of population shares per catchment, which was based on population density, assuming that highest treatment levels would apply to the most densely populated areas (Vigiak et al., 2018). The assumption may not hold everywhere, but this aspect is likely to play a secondary role.

Industrial Total Organic Carbon emissions reported in the *E*-PRTR totaled 0.8 M t/y, and were largely variable, both as total per country and amounts per inhabitant (Vigiak et al., 2018). Industrial emission loads reported by Norway were extremely high, more than five times larger than any other country; and emissions per capita were about 80 times larger than the average for all other countries. It is not clear if these data are reliable; they are larger than Williams et al. (2012) estimates (see also Supplementary material), which however were based on model assumptions. The large majority (>95%) of these emissions are coming from aquaculture and located in coastal areas. As they are not discharged in freshwater systems, there is no monitoring data to evaluate the reliability of these emissions. Lacking alternative information to assess the reliability of these emissions, they were retained in this study. When excluding Norway, TOC emissions averaged 1.1 kg/capita/y (median 0.54) but were very large (>5 kg/capita) in Finland and Sweden due to the importance of paper and forest industries, and in Italy (1.6 kg/capita) due to chemical, paper and wood industries. Industry emissions reported in the E-PRTR are however likely incomplete. First of all, only large facilities need to report in the register; further, reporting of TOC emissions is voluntary, thus the completeness of E-PRTR entries cannot be assessed (AMEC, 2014). Finally, the BOD/TOC rate changes with industrial type and waste treatment, but we assumed a constant ‘effective’ ratio established through calibration. As in previous studies (Williams et al., 2012), industrial emissions remain a large source of uncertainty of emissions to waters currently.

Emissions by livestock amounted to 28.6 M t/y of BOD at the source, and were thus the largest source of BOD to the environment. BOD abatement through either secondary treatment for intensive systems (94%) or basin retention for extensive systems (87–97%) were equally large, thus BOD to freshwater system was estimated at 1.8 M t/y, second source after point sources direct discharges (2.4 M t/y), confirming previous assessments of the importance of livestock for organic pollution in Europe (Malve et al., 2012; Voß et al., 2012; Wen et al., 2017). Livestock densities were taken from 2005 (Robinson et al., 2014), which was the latest available spatial dataset (an updated dataset has just been released by Gilbert et al., 2018, but at too coarse resolution for application at CCM2 scale). According to national statistics, livestock LUs have not changed sensibly from 2005 to 2015 across Europe (FAOSTAT, 2018), however some regional variations occurred, with a general decrease of cattle production in Northern Europe and an increase in pig production in Southern Europe. Large increases in cattle and pig densities occurred in The Netherlands, Serbia and Montenegro, and that of pigs in Albania. In Croatia cattle and sheep/goats production decreased but at the same time there was an increase in pigs and chicken production. Malta and Slovakia were in counter tendency, with reduction of livestock densities. Thus, from 2005 to 2015, a shift of livestock production densities toward more intensive systems would theoretically point to higher potential emissions from livestock sources to freshwater systems.

Emissions from urban wash-off was estimated at 1 M t/y, whereas from natural land at 0.6 M t/y. These were thus minor sources of BOD compared to point sources or livestock, however they were still important. Uncertainty in spatial data used to assess them are relatively minor, as both sources were based on a well-established spatial layer (CLC, 2012), with urban wash-off depending also on estimated mean annual precipitation.

### 4.3 Uncertainty in model parameters

While potential uncertainties in monitored data are to be acknowledged, the sheer number of data entries allowed for a balanced calibration of model parameters (Fig. 4, Fig. 5), albeit a spatial bias may stem from the heterogeneous data distribution in Europe (Fig. 2). Ten parameters may be considered many for a conceptual model, however initial ranges could be well defined with literature (Table 1) and low correlations between them (Table 2) indicated that parameters could be well identified without risk of over-parameterization of the model. Eventually, given the calibration results, it would be possible to simplify the model by adopting the same delay for both livestock and diffuse domestic sources, i.e. setting #LVST = #DD.

The calibrated parameter set mostly confirmed initial assumptions (Table 1). By fixing k_20_ at 0.56 the final distribution of BOD exponential decay rate after correction for water temperature k_T_ ranged from 0.29 to 0.49 across Europe, with mean value of 0.35/day. These values are very much in line with literature (Wen et al., 2017; Higashino and Stefan, 2017). A delay in travel time from diffuse domestic and livestock sources of 7 days (#DD, #LVST; Table 1) meant that basin retention was high, ranging from 87 to 97% of initial emissions, with a mean value of 92%. A delay of seven days is in good agreement with mean transit times in soils (McGuire and McDonnell, 2010).

The calibrated value for Eff.2 i.e. efficiency of secondary treatment, set at 94%, is high for literature and falls in the upper range of efficiencies calculated for a sample of WWTPs whose emissions are reported in the Waterbase-UWWTD database (Vigiak et al., 2018). Secondary treatment in WWTPs accounts for only 20% of connected not treated domestic waste, against 78.2% treated at tertiary treatment. The high sensitivity of Eff.2 was in part driven by the abatement for intensive livestock (Fig. 1). The calibrated Eff.2 is also high when compared to the global average of 85% set by Wen et al. (2017). Yet, a tendency of model to overestimate BOD in countries of high livestock densities, like Belgium or Denmark (Fig. 7), may indicate that BOD abatement for intensive livestock systems is still underestimated for European conditions, or conversely, that BOD emissions (400 g/LU/day) may be excessive. Model calibration however ensured that generally the importance of livestock or domestic emissions was not over estimated.

An industrial BOD/TOC ratio of 0.75 is consistent with ratios reported in literature for pulp and wood production (Grötzner et al., 2018), which is the majority of *E*-PRTR emissions to freshwaters. It may largely underestimate waste from aquaculture or other agro-industries, for which emission BOD/TOC ratio has been reported to be larger than two (Quayle et al., 2009; Kwarciak-Kozłowska and Bień, 2018), however agro-industries in the E-PRTR accounted for about 5% of TOC emissions, whereas aquaculture emissions are directly discharged to the sea and unmonitored therefore could not influence calibration. Improvements of model predictions could be achieved by reporting BOD emissions in the E-PRTR rather than TOC, as not only industry type but also technological treatment adopted by facilities largely influence BOD emissions.

EMC_U_, regulating BOD emissions of urban wash off, was possibly the most uncertain model parameter. The parameter was correlated to secondary and tertiary treatment efficiency (Table 2), however, different model responses of KGE and R^2^ (Fig. 4, Fig. 5) allowed to identify the parameter well. The calibrated parameter is in line with some literature (Rossman and Huber, 2016; Andrés-Doménech et al., 2018; Hur et al., 2018) but is high when compared to estimates by Williams et al. (2012), who considered this as part of discharge being treated in WWTPS. The median BOD load from urban wash-off from artificial land was about 4.5 t/km^2^, and accounted for 33% of urban waste (considering also WWTPS emissions). It is hard to assess how reliable this estimation is, as little is known about frequency and volumes of combined sewer overflows (CSO) or of BOD emissions in urban land net of WWTP discharges.

Conversely, natural land emissions are well in line with literature estimates (Aitkenhead and McDowell, 2000; Povilaitis, 2008; Shrestha et al., 2008). Overall, model parameterization of Table 1 could be considered robust. Future research should assess how model uncertainties will propagate on BOD emissions and fluxes spatially across Europe.

### 4.4 Change of BOD emissions from the 2000s

It may be interesting to compare BOD loadings change in time. Williams et al. (2012) assessed national BOD emissions for the year 2000 per sector and in total. As approaches between Williams et al. (2012) and this study differed for structure, parameters, and input data, comparison of emissions by sector is fraught by semantic and data uncertainty (an attempt to compare some aggregated figures is reported in Supplementary material). Instead, estimates of total BOD loads can be compared as both are results of model calibration at European scale.

**Fig. 10:**
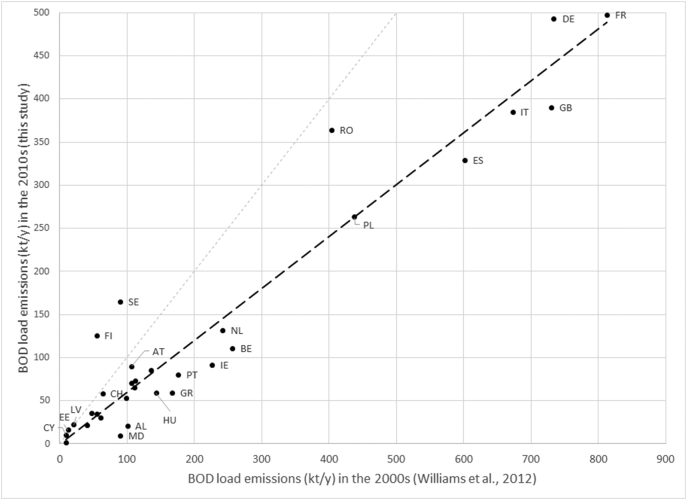
Total BOD emissions (kt/y) per country (32 countries) as estimated in this study (Y axis) for the 2010s and as estimated by Williams et al. (2012) for the 2000s (X axis). The grey dashed line indicates 1:1 relationship. The black dashed line indicates the regression line. Country codes are listed in Supplementary Material S1.

BOD emissions appear to have significantly declined in Europe since the 2000s (Fig. 10). Fitting a linear regression of country BOD emissions in 2010s (this study) as function of those in 2000 (Williams et al., 2012) results in a coefficient of 0.6 (adjR^2^ = 0.92; sample size = 32, excluding Norway for which industrial BOD emissions in this study are much larger than previously estimated, see Supplementary material). Large reductions (>100 kt/y) in BOD emissions occurred in Great Britain, France, Italy, Spain, Germany, Poland, Belgium, Ireland, the Netherlands and Greece. Conversely, BOD fluxes of this study for a few Nordic countries (Sweden, Finland, Estonia, and Norway) were larger than in Williams et al. (2012), likely because in this study natural area emissions were included in the model, whereas these were absent from 2000s assessment.

Current BOD fluxes have thus declined in time, especially thanks to improvements in wastewater treatments (EEA, 2015; Worrall et al., 2018). Decline in domestic waste emissions can be estimated at about 30% (see Supplementary material S3). Currently, in the European continent analyzed in this study, about 62% of domestic waste is treated at tertiary level. Within the EU28 this share raises to 68%. Further abatement of BOD from domestic waste could be gained by treating domestic waste that is connected but not yet treated, or upgrading primary treatments to secondary or tertiary level. In EU28 these sources represent <5% of domestic waste emissions, but in some countries domestic waste is still an important source of BOD.

Water quality due organic pollution was assessed to be in moderate to poor conditions (BOD concentrations >5 mg O_2_/L) in about 12% of freshwater systems (14% of EU28 freshwaters). While reaching full compliance of UWWTD directive will certainly further improve freshwater quality, other management strategies should also be considered. In this assessment, industrial emissions were an important source of BOD loads to European freshwater and the seas. Regulation or reduction of intensive livestock production systems will allow tackling the major source of BOD emissions in agricultural areas. In urban catchments, management of urban wash off may probably reduce BOD effectively. Last but not least, the reduction of streamflow, and thus of BOD dilution capacity, due to increasing water demands or climate change, may exacerbate poor habitat conditions, particularly in water scarce areas. The current model may help exploring the likely impact of these scenarios at the European scale.

## 5 Conclusions

A conceptual model for assessing current BOD fluxes as a proxy of organic pollution and water quality across Europe was developed based on the most updated European-wide datasets. In particular, estimation of domestic waste benefitted of detailed information of waste generated and treated in wastewater treatment plans. The model accounted for urban wash-off and natural area emissions in addition to major anthropogenic sources such as domestic, industrial and livestock waste. The uncertainty in some sources of BOD still remained important, especially that of industrial emissions or urban wash-off.

Acknowledging uncertainty in sources and hydrological pathways, ten model parameters were calibrated at 2157 BOD monitoring stations. This allowed for robust characterization of the model, yielding a final parameter set that mostly confirmed literature values. Model mean absolute errors were 1.2 mg O_2_/L and 0.4 t/km^2^ for the validation dataset of 1134 stations, and were acceptable in consideration of the many sources of uncertainty affecting monitoring network datasets and the resolution and accuracy of input data. The model could correctly classify reaches for BOD concentrations classes in 69% of cases. Conversely, high overestimations (incorrect classification by 2 or more classes) were 2% and large underestimations were 5% of cases. The model was thus judged fit to assess current mean conditions across Europe, albeit errors in detecting high BOD concentrations remained high. Potential improvement in the model could be achieved by (i) improving input datasets, for example industrial BOD emissions; (ii) enlarging the BOD monitoring dataset, especially in regions currently under or not represented; and (iii) coupling the model to a more dynamic hydrological model to take into consideration the impact of annual streamflow changes on BOD concentrations. Importantly, given uncertainty in inputs and outputs highlighted by the analysis, further research should aim at assessing model uncertainty in BOD fluxes spatially.

About 262,000 km (12%) of freshwater network (14%, 210,000 km in the EU28 zone) were assessed as being in moderate to poor conditions due to organic pollution (BOD concentrations > 5 mg O_2_/L). Dominant sources of BOD emissions were point sources and intensive livestock systems. Improving wastewater treatment may reduce BOD loads effectively, especially in Eastern Europe. Compliance of regulative efforts set by the UWWTD Directive are however close to reach their maximum impact. Regulation and reduction of industrial emissions may abate BOD loadings to the seas effectively, especially if targeting emissions in coastal areas. Thus while current legislative regulations may still improve conditions further, other management strategies should also be considered to reach or maintain the good ecological status of freshwater systems as required by the WFD. The conceptual model can help exploring the effectiveness of potential alternative management at the European scale.

## Appendix A Supplementary data

Supplementary material Image 1

## Footnotes

1 Under United Nations Security Council Resolution 1244/99.
