# Supplementary material for: Predicting biochemical oxygen demand in European freshwater bodies
Source: Sci Total Environ. 2019 May 20;666:1089–105. doi: 10.1016/j.scitotenv.2019.02.252 (PMC6451040; doi:10.1016/j.scitotenv.2019.02.252)
Supplement: Supplementary file 1 — Supplementary material [file mmc1.pdf]

Supplementary Material to

‘Predicting Biochemical Oxygen Demand in European freshwater bodies’

Olga Vigiak, Bruna Grizzetti, Angel Udias-Moinelo, Michela Zanni, Chiara Dorati, Fayçal Bouraoui,

Alberto Pistocchi.

Content:

S.1 Distribution of model parameters in behavioral parameter sets

Table S1. Country code and total BOD load inputs (t/y) estimated in this study: DOM_PS = BOD from domestic point sources; DOM_DIF = BOD from diffuse domestic sources; IND = BOD from industries; LVST_HD = BOD from high density livestock facilities; LVST_LD = BOD from lowh density livestock (diffuse); Urb = BOD from Urban areas; FRST = BOD from natural areas; Total = sum of BOD input sources.

S.2 Model evaluation in four Globaqua basins

S.3 Comparison of BOD emissions in 2000s (Williams et al., 2012) and in 2010s (this study)

S.1 Distribution of model parameters in behavioral parameter sets

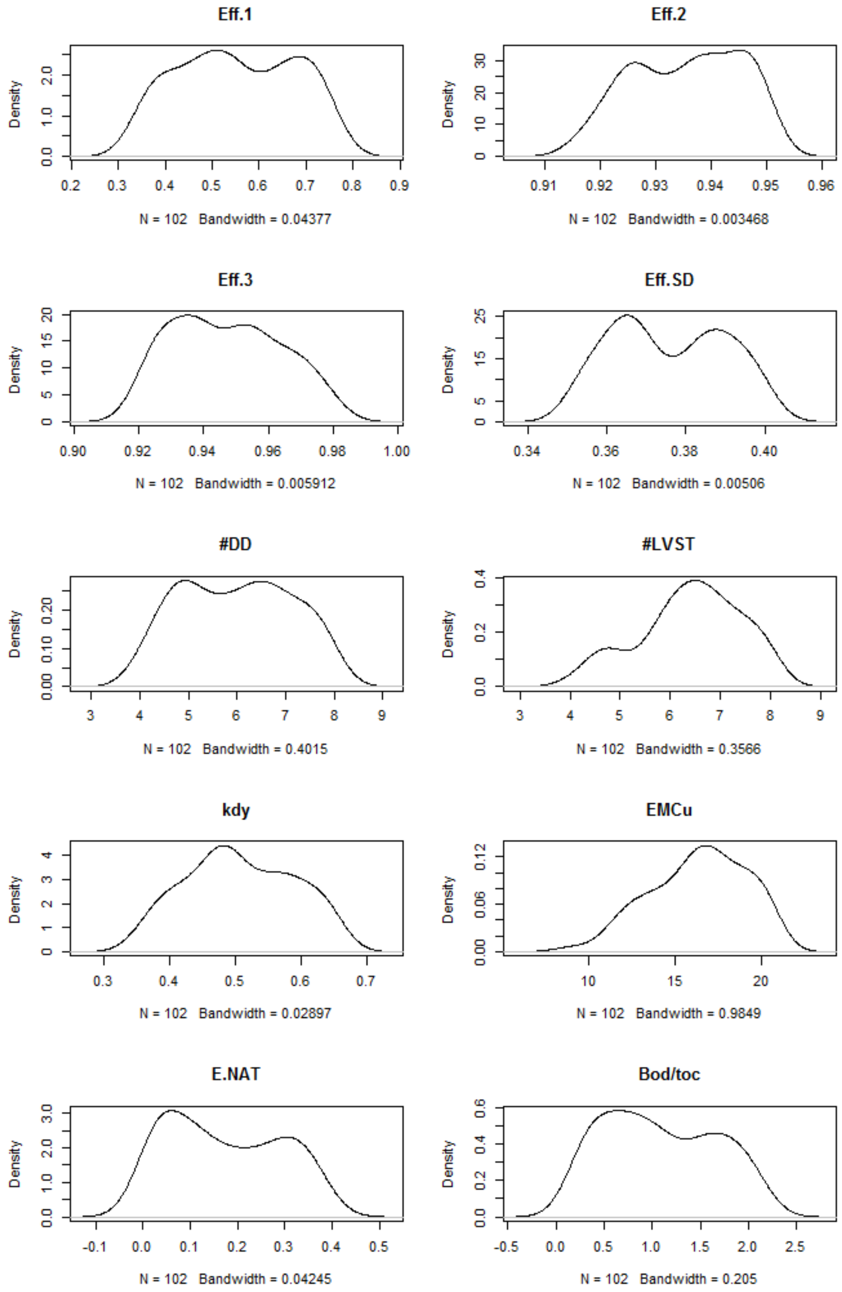

Figure S1. Distribution of model parameters of 102 behavioral parameter sets.

Behavioral parameter distributions are unimodal or bimodal, due to some correlations among parameters. In most cases, behavioural parameter ranges remained large, indicating potential important uncertainty in model estimations.

Table S1. Country code and total BOD load inputs (t/y) estimated in this study: DOM_PS = BOD from domestic point sources; DOM_DIF = BOD from diffuse domestic sources; IND = BOD from industries; LVST_HD = BOD from high density livestock facilities; LVST_LD = BOD from lowh density livestock (diffuse); Urb = BOD from Urban areas; FRST = BOD from natural areas; Total = sum of BOD input sources.

Country	CODE	DOM_PS	DOM_DIF	IND	LVST_HD	LVST_LD	Urb	Frst	Total
Andorra	AD	129	2	0	9	314	1	57	512
Albania	AL	4720	1412	0	3702	70363	4343	2665	87205
Austria	AT	17860	0	8820	21961	121791	22216	7879	200527
Bosnia Herzegovina	BA	30500	2001	0	5379	83592	4284	4758	130514
Belgium	BE	30153	897	4671	45736	10082	26917	1701	120157
Bulgaria	BG	43761	454	2007	4833	103935	18363	9328	182681
Belarus	BY	16450	435	0	16474	128111	34	7109	168613
Switzerland	CH	15009	2	1892	17969	35021	16678	3790	90361
Cyprus	CY	5400	151	0	1834	17090	1476	1144	27095
Czech Republic	CZ	17776	2114	1900	12908	140343	18743	5612	199396
Germany	DE	97883	1021	19768	181659	402788	138123	24604	865846
Denmark	DK	10192	0	1717	46005	36339	10301	2071	106625
Estonia	EE	1558	1	502	1278	35639	3796	5564	48338
Spain	ES	88217	49	9172	113812	757817	39209	38715	1046991
Finland	FI	4733	1261	33690	4455	139218	16514	49887	249758
France	FR	66240	4847	22924	208641	545539	124080	36076	1008347
Great Britain	GB	81643	220	55801	131009	420763	75674	12633	777743
Guernsey	GG	82	0	0	6	121	54	9	272
Greece	GR	9515	939	433	10888	238109	12382	12531	284797
Croatia	HR	40126	167	0	5500	53917	9468	6558	115736
Hungary	HU	11253	1265	2236	14153	121348	17888	4887	173030
Ireland	IE	8586	355	53	64520	76573	7967	3980	162034
Isle of Man	IM	125	0	0	438	392	152	86	1193

Italy	IT	105760	1651	70831	87687	344307	77617	22673	710526
Jersey	JE	115	0	0	8	292	66	17	498
Kosovo*	XS	24402	759	0	446	17606	1518	1085	45816
Lichtenstein	LI	42	1	0	54	253	147	18	515
Lithuania	LT	2222	928	0	4795	101753	8114	5399	123211
Luxemburg	LU	774	13	139	1923	621	1155	186	4811
Latvia	LV	3341	753	0	1659	56995	4345	7065	74158
Monte Carlo	MC	0	0	0	0	0	6	0	6
Moldova	MD	4963	840	0	2072	23046	130	261	31312
Montenegro	ME	4465	276	0	176	17727	1966	1918	26528
Macedonia, FYR	MK	25876	686	0	1557	38625	1505	2192	70441
Malta	MT	461	0	354	430	342	277	18	1882
Netherlands	NL	15592	87	6443	83779	26803	22511	1805	157020
Norway	NO	22046	759	312986	3532	154976	15284	49745	559328
Poland	PL	48414	7756	6073	88942	362560	61983	21006	596734
Portugal	PT	25280	142	6628	19557	127019	15051	8243	201920
Romania	RO	235999	576	2777	31964	483293	43996	14183	812788
Serbia	RS	80250	2251	2541	14842	138343	9882	5678	253787
Russian Federation	RU	53001	3592	0	6358	67186	77	108502	238716
Sweden	SE	11519	2	39162	6345	220122	21443	65994	364587
Slovenia	SI	7754	772	340	4553	21429	4081	2372	41301
Slovak Republic	SK	4836	2303	2414	4413	71852	11767	4167	101752
San Marino	SM	398	1	0	1	102	39	2	543
Turkey	TR	508968	4797	0	61849	890525	38378	39356	1543873
Ukraine	UA	22170	2602	0	4122	82764	246	3149	115053

* under United Nations Security Council Resolution 1244/99

S.2 Model evaluation in four Globaqua basins

An independent BOD monitored dataset was provided in four European basins within the Globaqua project (Navarro-Ortega et al., 2015): the Adige, the Ebro, the Evrotas and the Sava (Fig. S2 indicates their location in relation to the European dataset). The basins encompass a range of European geographic and socio-ecological conditions. The Adige (about 12,000 km^2) is an alpine basin located in the north-eastern part of Italy. BOD trends in the basin are mainly correlated to population and tourism activities (Diamantini et al., 2018). The Ebro River drains a basin of 85,550 km^2 in Spain. The basin hosts more than 2.7 million inhabitants and approximately 45% of the population concentrate in five cities located next to the Ebro River or its tributaries. BOD concentrations in the river are also affected by agriculture and livestock production and are exacerbated by water abstractions for agricultural and industrial activities that reduce river dilution capacity (Diamantini et al. 2018). The Evrotas river basin covers an area of 1,739 km^2 in the Peloponnese (Greece). The Evrotas River is about 90 km in length and is fed by numerous intermittent and ephemeral tributaries. The basin has a typical Mediterranean climate. The population of the basin is about 45,000 inhabitants. Water quality is affected by high water abstraction for irrigation, disposal of agro-industrial wastes, and agrochemical pollution (Skoulikidis et al., 2011). The Sava River is the largest tributary of the Danube River and its basin extends over almost 100,000 km^2 across Slovenia, Croatia, Bosnia and Herzegovina, Serbia, Montenegro, and Albania (ISRBC, 2009). BOD trends are correlated to population (Diamantini et al., 2018), which amounts to around 8.2 million (ISBRC, 2009).

The BOD dataset of Globaqua (GLOBAQUA, 2017) is quite heterogeneous for number of stations, observation period, and frequency of monitoring. In consideration of European temporal BOD trends, only data from 2008 onward were retained in the analysis. BOD was monitored in the basins with frequency varying from episodic sampling campaigns (in the Ebro and Evrotas) to monthly (in the Sava and Adige). All BOD observations per station were averaged, and only stations with at

least 3 observations and encompassing at least two years were kept for comparison with model outputs. This sample size is very small, but allowed retaining at least a couple of stations in the Evrotas River where BOD had only been measured during water quality research campaigns.

The dataset was used to assess model performance in specific basins. The Adige and the Sava basins were part of the calibration dataset of the model, and some stations are in common between the Globaqua and the European datasets, while the Ebro and Sava were part of the validation dataset, while no data was available for Greece in the Waterbase Rivers dataset.

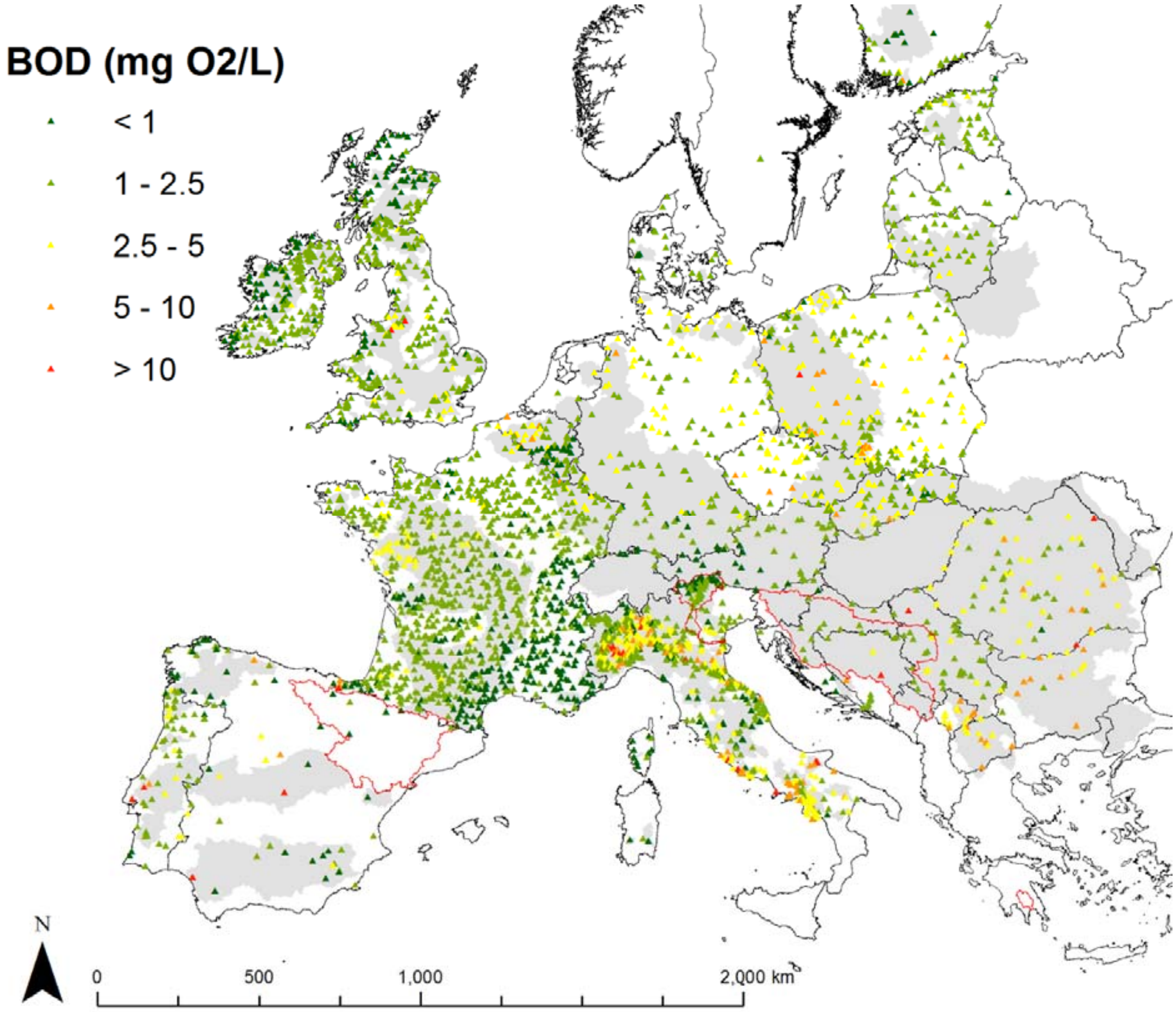

Figure S2. Mean annual BOD concentration [mg O_2/L] monitored data used in this study. Grey background show basins whose data was used for model calibration. The outlines of four GLOBAQUA basins are shown in red.

S.2.2 Model results in the Globaqua basins

Modelled BOD concentrations for the four European basins are shown against monitored data of the Waterbase Rivers dataset and of Globaqua dataset in Fig. S3. In the Adige, the model generally under predicted monitored concentrations, especially for the Globaqua dataset. In the Ebro, Waterbase River data were well predicted whereas BOD in Globaqua stations was mostly under predicted. In the Evrotas modelled concentrations were remarkably close to monitored values.

In the Sava, a large scatter was observed for the Waterbase River dataset, especially for three stations. One of these station (RS_RV_45098, which is omitted from figure) had a very large predicted concentration (above 40 mg/L). It is likely that this station is misplaced or that streamflow is incorrect. This station is in an urban area and channel regulation may change hydrology considerably. The mean annual streamflow estimated for this location is 0.7 m^3/s while BOD loadings are very high because this station is part of Belgrade town; unfortunately, no information on streamflow for this station is available in the Waterbase Rivers dataset to help checking its location or streamflow estimation. This data entry is thus omitted in the evaluation of model results.

While errors for the Globaqua dataset were generally larger than for Waterbase Rivers, mean error statistics for the basins (Table S2) were close to European means (Table 3 in paper).

Table S2. Sample size (number of stations), mean absolute error (MAE) and root mean square error (RMSE) of BOD modelled concentration (mg/L) in four European basins for the Waterbase Rivers dataset and an independent dataset (Globaqua project).

	Waterbase Rivers dataset			Globaqua dataset		
	Sample size	MAE	RMSE	Sample size	MAE	RMSE
Adige	18	0.67	0.78	28	1.32	1.50
Ebro	3	0.53	0.83	18	1.90	2.29
Evrotas	0	NA	NA	3	0.61	0.65
Sava	20*	1.18	1.56	17	1.17	1.24

* Station RS_RV_45098 is omitted because of a likely location error. By including it, MAE in the Sava would increase to 3.09, RMSE to 9.14.

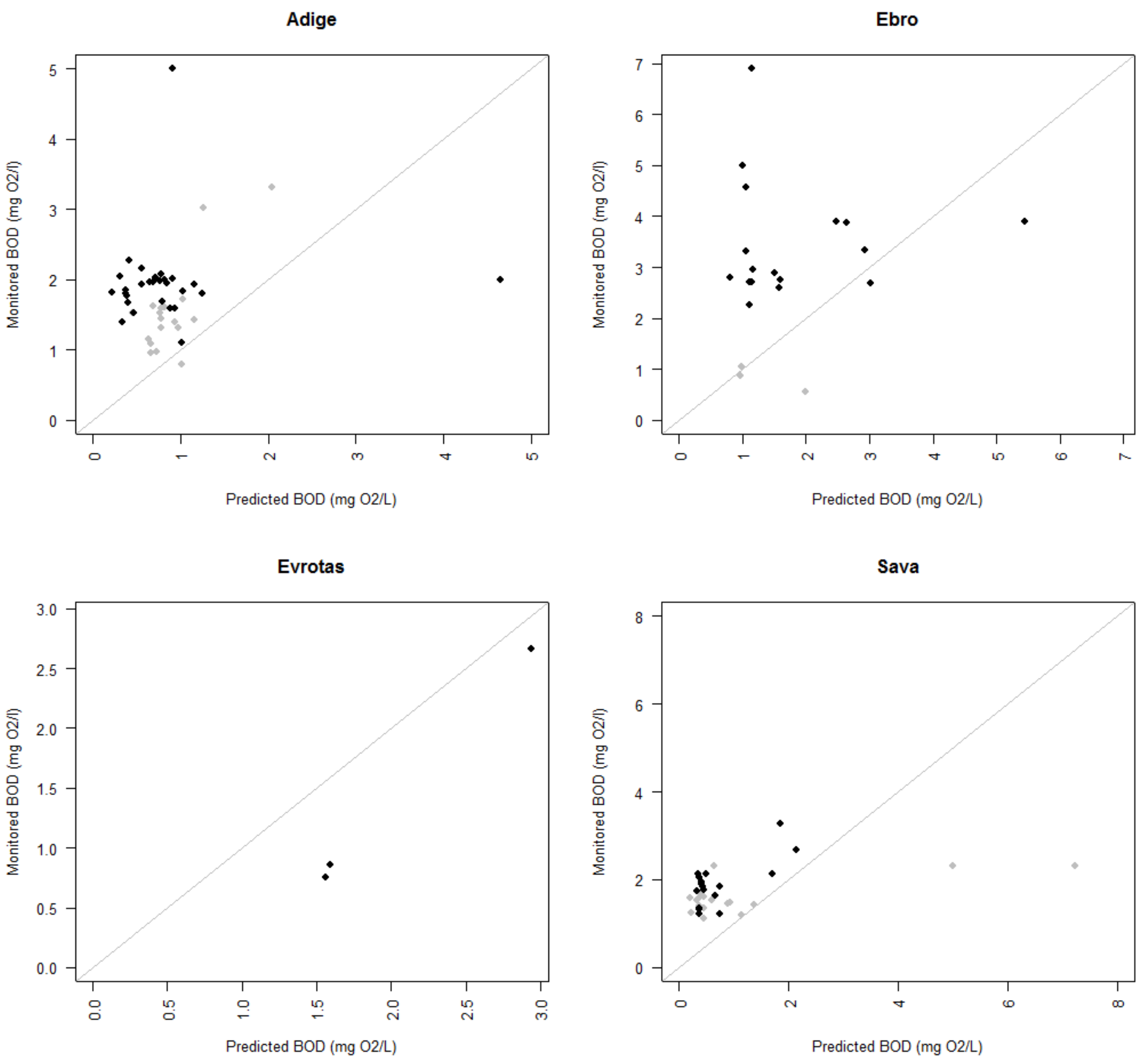

Figure S3. Modelled and monitored BOD concentration in four European basins for which an independent dataset (Globaqua project) was available. Grey dots indicate Waterbase River stations data; black dots indicate Globaqua dataset. Grey line indicates 1:1 relationship.

The availability of two datasets offer an opportunity to compare observed stations/year data entries that were common in the Waterbase Rivers and the Globaqua datasets for the Adige and Sava basins (Fig. S4). While in most cases there was good correspondence between reported values, large discrepancies could be observed between data entries for 2012 in the Adige and for one station in the Sava (HR_RV_14001 of Waterbase Rivers; Upstream Una Jasenovac in Globaqua dataset) for which Waterbase Rivers indicated lower BOD concentrations than in Globaqua dataset.

Discrepancies in the Adige basin for 2012 were larger than 1 mg/L and for the Sava station of about 0.4 mg/L. These inconsistencies are large in relation to mean BOD values.

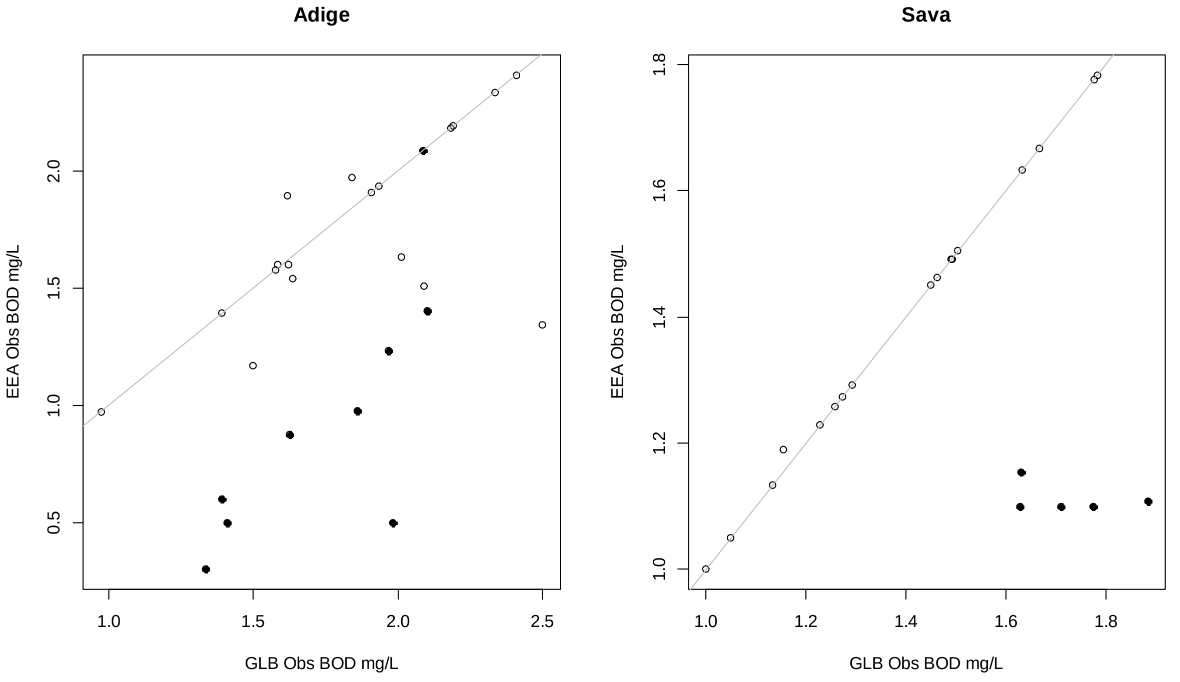

Figure S4. Comparison of monitored data in Waterbase Rivers database (y axis) and in Globaqua dataset (x axis) for common stations and years. In the Adige (left) black dots indicate data entries for 2012. In the Sava (right) dot plots refer to station (HR_RV_14001 of Waterbase Rivers; Upstream Una Jasenovac of Globaqua dataset).

S.3 Comparison of BOD emissions in 2000s and this study

Williams et al. (2012) published national BOD emissions estimated for the year 2000 for 33 European countries. The dataset offers an opportunity to compare emissions between the two periods, however data inputs and estimation methods are very different, hampering direct comparison of sources, instead some sources were aggregated (Table S3). In particular, (i) urban runoff was added to domestic sources as Williams et al. considered urban wash-off treated in WWTPs; and (ii) livestock sources in Williams et al. (2012) were estimated with a statistical approach (Malve et al., 2012) and that study did not consider natural area emissions, so livestock and natural emissions were aggregated.

Table S3. Aggregation of BOD sources in the two study for comparison

Source of waste	Williams et al. 2012	This study
Domestic+Urban	Domestic, scattered dwellings, and urban runoff	Domestic (point and diffuse), and urban wash-off
Industrial	Manufacturing	Industrial
Other diffuse sources	Livestock	Livestock and natural areas

Figures S5 to S7 shows the country comparison for the three aggregated sources of emissions. In Fig. S5 a considerable reduction, of about 30%, of domestic (plus urban wash-off) emissions is estimated for most countries. The largest exceptions occurred in RO, where an increase in emissions is due to a high share of connected and not treated domestic waste (Vigiak et al., 2018), and in FR and DE, where urban wash-off is considered to contribute large emissions of BOD in our study.

Industrial emissions (S6) in the two studies differed very much, particularly for NO, for which very high BOD loads were derived from E-PRTR dataset. It is unclear if these industrial emissions are correct, however they were retained in the study.

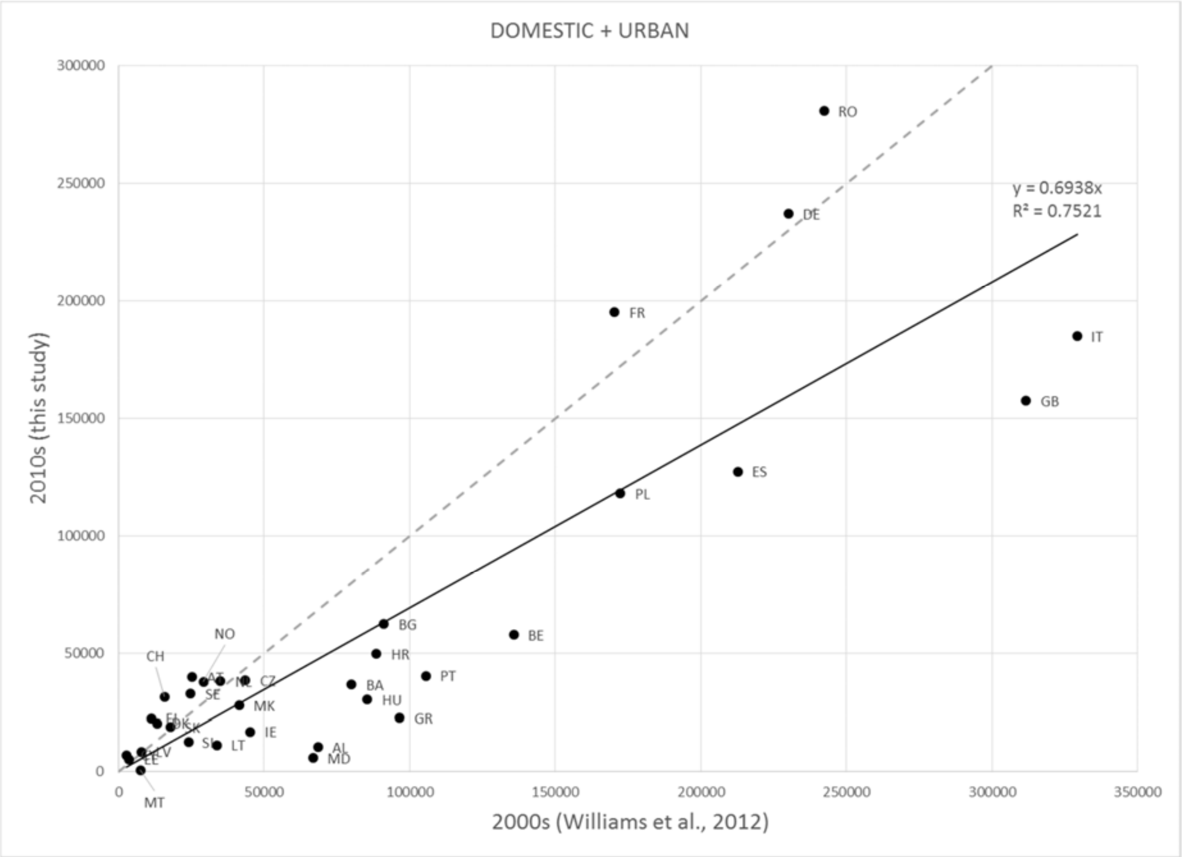

Figure S5. Country domestic and urban BOD emissions (t/y) estimated for the 2000s (Williams et al., 2012; x axis) and in the 2010s (this study, y axis). The black line indicates linear regression equation; the dashed grey line indicates 1:1 relationship.

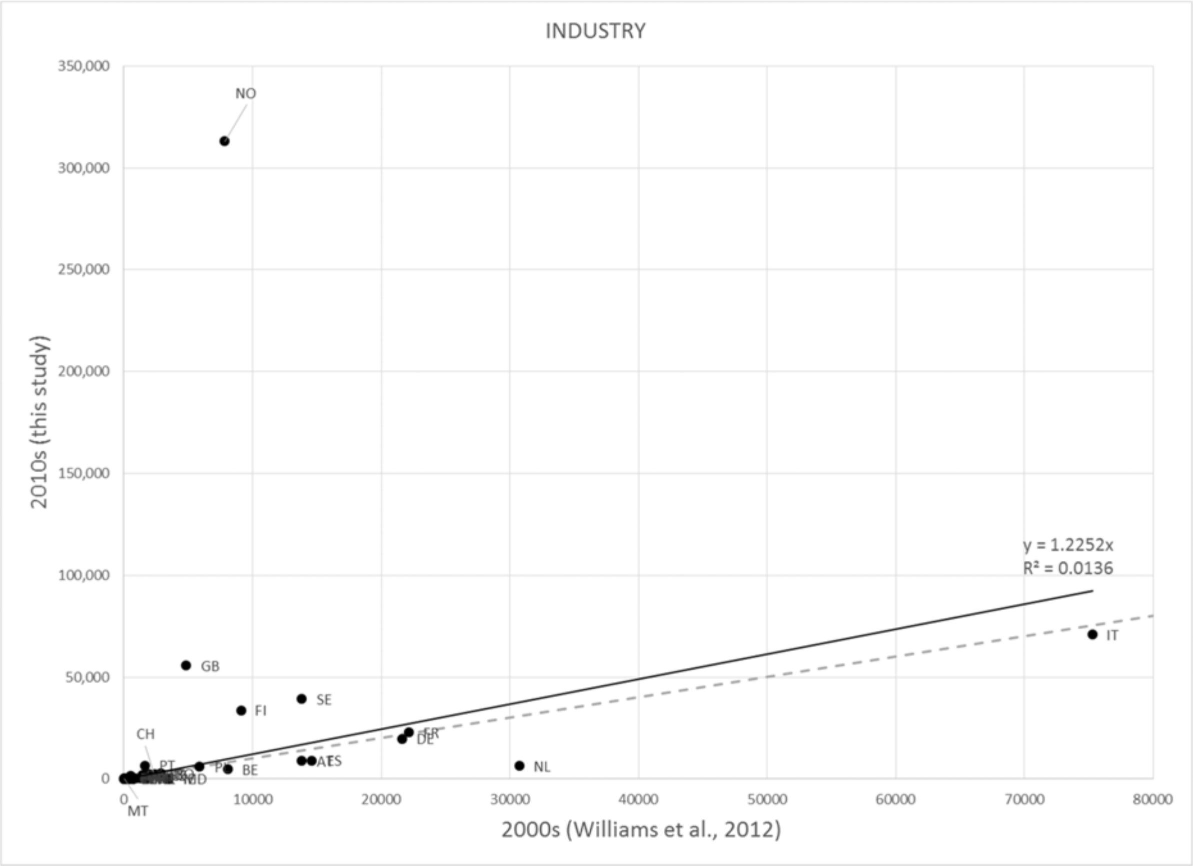

Figure S6. Country industrial BOD emissions (t/y) estimated for the 2000s (Williams et al., 2012; x axis) and in the 2010s (this study, y axis). The black line indicates linear regression equation; the dashed grey line indicates 1:1 relationship.

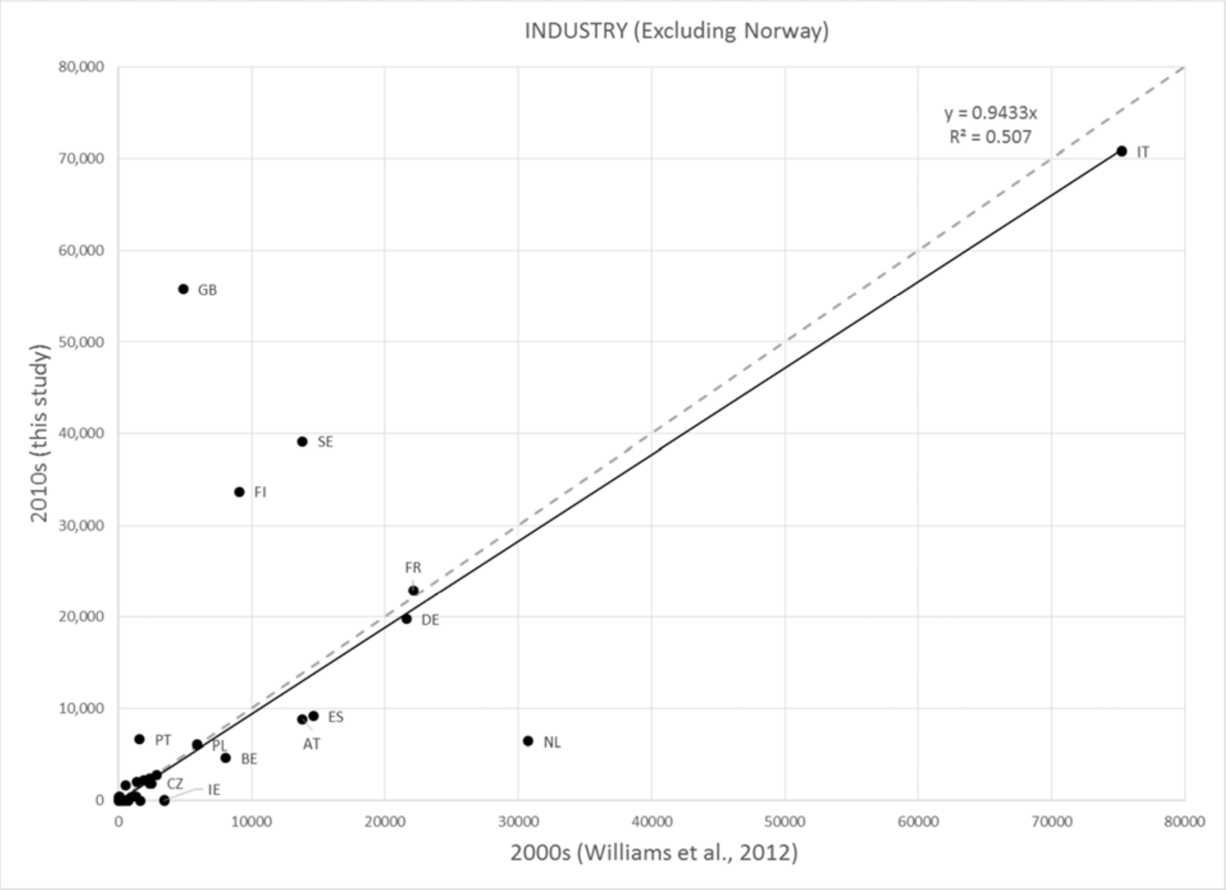

Figure S6b. Country industrial BOD emissions (t/y) estimated for the 2000s (Williams et al., 2012; x axis) and in the 2010s (this study, y axis) when excluding Norway. The black line indicates linear regression equation; the dashed grey line indicates 1:1 relationship.

When excluding Norway, industrial emissions in this study were 94% what estimated in Williams et al. (2012) (Figure S6b). Yet industrial emissions in this study are likely incomplete as for many countries, also within EU28 (CY, HR, LV, LT), BOD industrial emissions were null.

Other diffuse sources estimated in our study were about half those estimated in Williams et al. (2012). In both studies, these sources are heavily calibrated on available data and depending on other sources so a compensation effect in diffuse sources can be expected.

Finally, figure S9 shows total emissions estimated in the two studies (including Norway). The reduction in BOD total emissions between 200s and 2010s is estimated at about 32%.

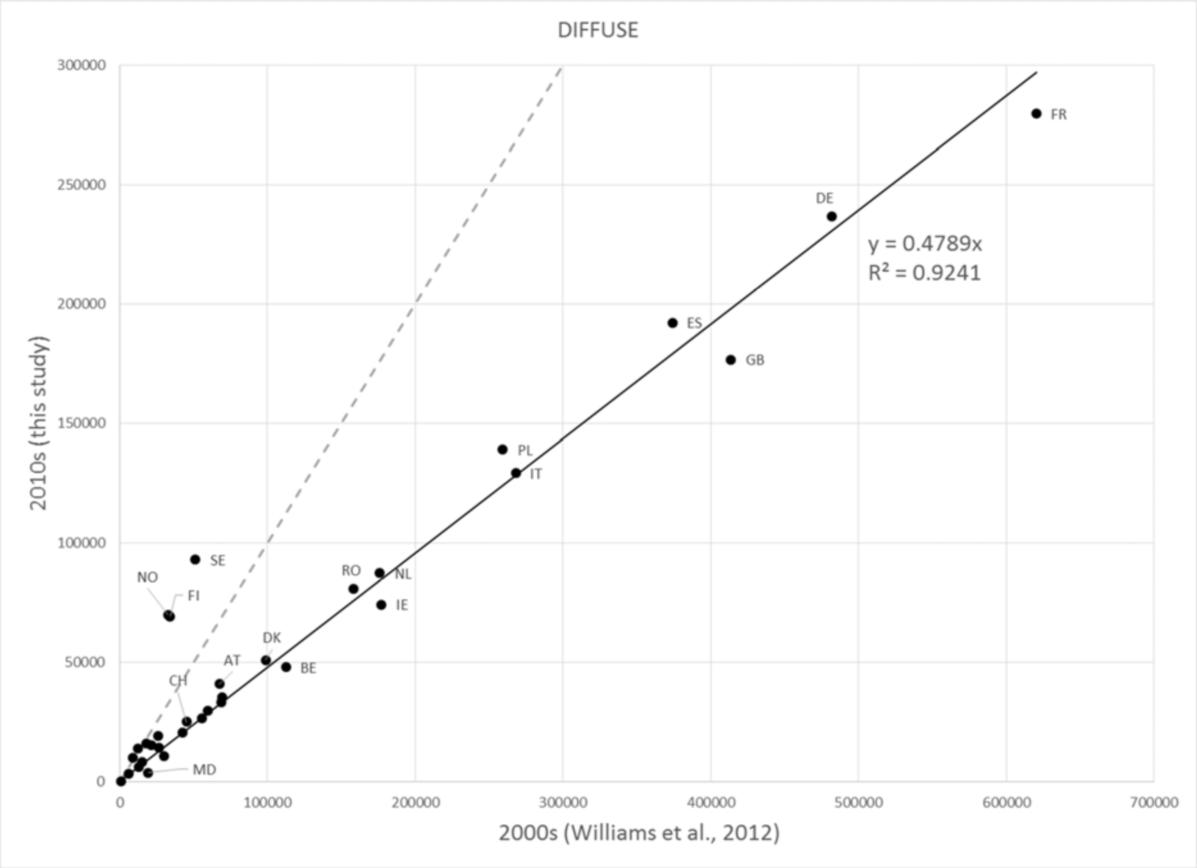

Figure S7. Country diffuse (other than domestic) BOD emissions (t/y) estimated for the 2000s (Williams et al., 2012; x axis) and in the 2010s (this study, y axis). The black line indicates linear regression equation; the dashed grey line indicates 1:1 relationship.

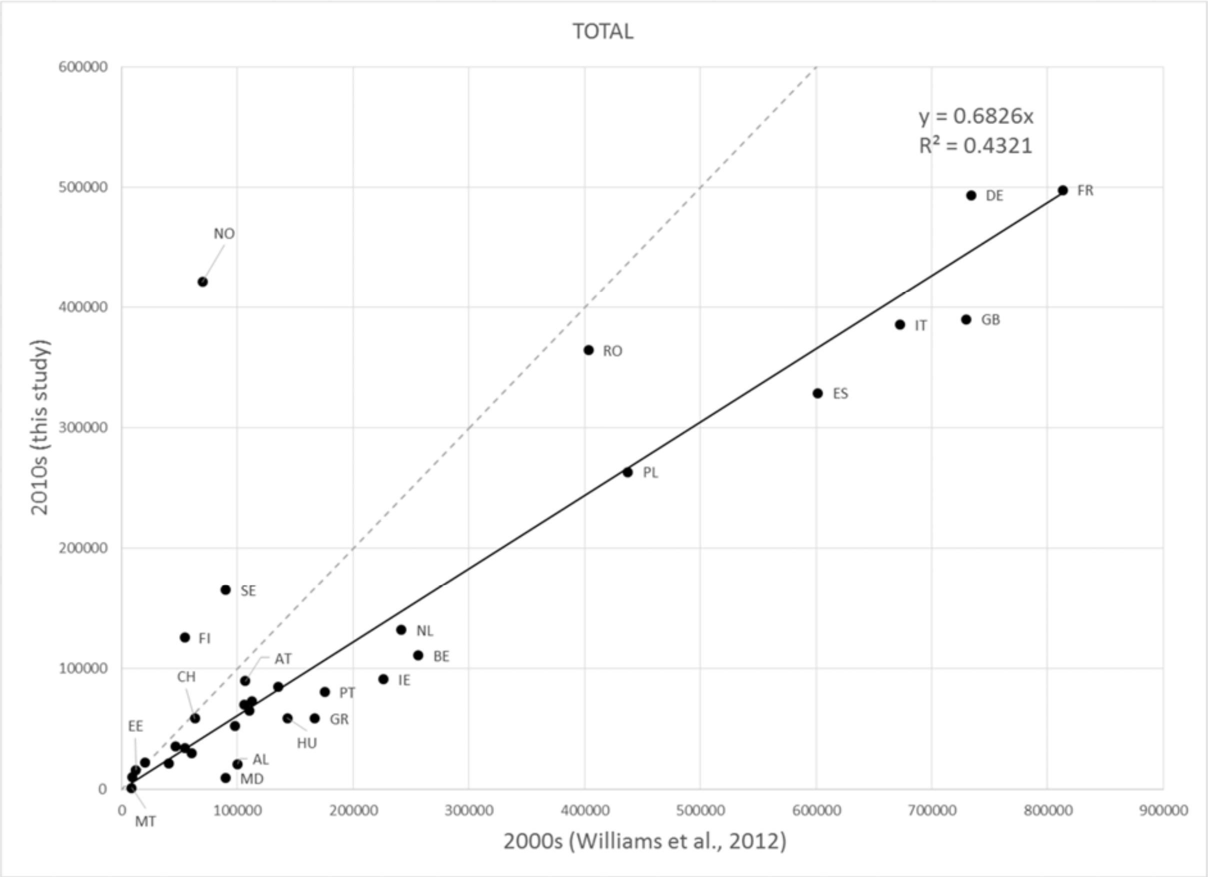

Figure S8. Country total BOD emissions (t/y) estimated for the 2000s (Williams et al., 2012; x axis) and in the 2010s (this study, y axis). The black line indicates linear regression equation; the dashed grey line indicates 1:1 relationship.

Further references to Supplementary Material

Diamantini, E., Lutz, S.R., Mallucci, S., Majone, B., Merz, R., Bellin, A. 2018. Driver detection of water quality trends in three large European river basins. Science of the Total Environment 612, 49-62; doi: 10.1016/j.scitotenv.2017.08.172

Navarro-Ortega, A., Acuña, V., Bellin, A., Burek, P., Cassiani, G., Choukr-Allah, R., Dolédec, S., Elosegi, A., Ferrari, F., Ginebreda, A., Grathwohl, P., Jones, C., Rault, P.K., Kok, K., Koundouri, P., Ludwig, R.P., Merz, R., Milacic, R., Muñoz, I., Nikulin, G., Paniconi, C., Paunović, M., Petrovic, M., Sabater, L., Sabater, S., Skoulikidis, N.T., Slob, A., Teutsch, G., Voulvoulis, N., Barceló, D. 2015. Managing the effects of multiple stressors on aquatic ecosystems under water scarcity. The GLOBAQUA project. Sci. Total Environ. 503 (504), 3–9.

Skoulikidis N, Vardakas L, Karaouzas I, Economou A, Dimitriou E, Zogaris S. 2011. Assessing water stress in Mediterranean lotic systems: Insights from an artificially intermittent river in Greece. Aquatic Sciences, Special Issue: Recent Perspectives on Temporary River Ecology 73: 581–597
